# SCL15 Promotes Seed Longevity Acquisition in *Arabidopsis thaliana* by Enhancing Antioxidant and Repair Mechanisms During Maturation

**DOI:** 10.1111/ppl.70907

**Published:** 2026-05-06

**Authors:** Ming‐Jun Gao, Cathy Coutu, Qi Chen, Myrtle Harrington, Rong Zhou, Dwayne Hegedus

**Affiliations:** ^1^ Agriculture and Agri‐Food Canada, Saskatoon Research Centre Saskatoon Saskatchewan Canada; ^2^ State Key Laboratory of Tea Plant Biology and Utilization Anhui Agricultural University Hefei Anhui China

**Keywords:** desiccation tolerance, longevity, oxidative stress, reactive oxygen species, seed vigor

## Abstract

To ensure seed longevity and successful germination, orthodox seeds acquire protective and repair mechanisms during the late stage of seed development to counteract the detrimental effects of desiccation and subsequent rehydration, processes often associated with oxidative stress. SCARECROW‐LIKE 15 (SCL15) was identified as a key regulator of seed longevity acquisition through transcriptomic profiling of maturing seeds from *Arabidopsis thaliana* wild type (Col‐0), an *SCL15* mutant, and transgenic lines overexpressing *SCL15* under the seed‐specific *Napin* promoter. *SCL15* influences pathways associated with reactive oxygen species (ROS) detoxification, chlorophyll degradation, DNA and protein repair, and the accumulation of protective molecules, including late embryo abundant proteins, heat shock proteins, raffinose family oligosaccharides, and 12S globulins during seed maturation. Loss of *SCL15* function impaired seed vigor and storability, accompanied by elevated ROS levels, chlorophyll retention, and reduced antioxidant capacity during seed maturation and dry storage. In contrast, *SCL15* overexpression enhanced antioxidant activity, chlorophyll breakdown, and the accumulation of protective and repair factors. Together, these findings indicate that SCL15 contributes to maintaining seed viability by coordinating antioxidant defenses with protective and reparative systems during seed maturation, thereby promoting desiccation tolerance and the acquisition of longevity. In addition, the present results, together with our previous work, suggest that SCL15 may participate in integrating hormonal and circadian pathways to balance dormancy release with the acquisition of seed longevity.

## 1 Introduction

Seed longevity, the capacity of seeds to remain viable over extended periods, is vital for biodiversity conservation, crop productivity, and ecological resilience. However, seed aging is an inevitable and irreversible process driven by cumulative biochemical damage during storage. Central to this decline is oxidative stress resulting from an imbalance between the generation of reactive oxygen species (ROS) and the antioxidant capacity of the seed, leading to damage of lipids, proteins, nucleic acids, and membranes (Sano et al. 2016; Zhou et al. 2020). While low levels of ROS play regulatory roles in seed development and germination, excessive ROS accumulation is a key factor limiting seed longevity (McDonald 1999; Bailly et al. 2008; Kranner et al. 2010). ROS‐induced damage includes oxidation of methionine residues (Stadtman 2006) and DNA strand breaks (Bray and West 2005), compromising seed viability. ROS are produced not only during dry storage and imbibition, but also accumulate during late seed maturation when desiccation tolerance is established. This necessitates a tightly regulated network of antioxidant, protective, and repair pathways to ensure seed survival.

To counteract desiccation‐ and ROS‐induced damage, orthodox seeds acquire a suite of protective mechanisms during maturation. These include accumulation of late embryo abundant (LEA) proteins, small heat shock proteins (HSPs), soluble sugars, and antioxidants, alongside the formation of a glassy cytoplasm and the dismantling of photosynthetic components to reduce photooxidative stress (Wehmeyer and Vierling 2000; Berjak and Pammenter 2013). Additionally, seeds employ damage repair mechanisms upon imbibition, such as PROTEIN L‐ISOASPARTYL METHYLTRANSFERASE (PIMT), which restores function to isoaspartyl‐modified proteins, including key regulatory transcription factors like ABA INSENSITIVE 3 (ABI3) and ABI5 (Ogé et al. 2008; Kamble and Majee 2020, 2022; Kamble et al. 2024).

Seed longevity is acquired during the late maturation phase, after storage reserve accumulation and before desiccation‐induced dormancy is fully established (Righetti et al. 2015; Leprince et al. 2017). Yet, the regulatory landscape governing this acquisition remains poorly defined. While transcription factors such as ABI3, ABI5, WRKYs, and members of the LAFL (LEAFY COTYLEDON1 [LEC1], ABSCISIC ACID INSENSITIVE3 [ABI3], FUSCA3 [FUS3], and LEC2) network are known to contribute to seed maturation and longevity (Righetti et al. 2015; Zinsmeister et al. 2016; Ramtekey et al. 2022), many upstream integrators remain unidentified.

SCARECROW‐LIKE15 (SCL15), an eudicot‐specific member of the GIBBERELLIC‐ACID INSENSITIVE, REPRESSOR of GAI and SCARECROW (GRAS) transcription factor family (Tian et al. 2004), was previously shown to suppress embryonic gene expression during germination through interaction with HISTONE DEACETYLASE19 (HDA19) (Gao et al. 2015) and to act as an auxin‐responsive regulator in *Brassica napus* (Gao et al. 2004). SCL15 is expressed in vascular tissues and localized in phloem companion cells of the ovule funiculus (Gao et al. 2015). Moreover, mutation of *SCL15* causes markedly reduced expression of seed maturation genes *ABI3*, *CRA1* (Gao et al. 2015) and *ABI5* (Gao et al. 2025) in developing seed, especially at the late stage of seed development, suggesting a role for SCL15 in seed maturation processes. Recently, it was linked to the regulation of seed dormancy release by coordinating hormonal and circadian cues (Gao et al. 2025). In this study, we identified SCL15 as a positive regulator of seed longevity in *Arabidopsis thaliana* . Through transcriptomic, physiological, and biochemical analyses of *scl15‐1* mutants and *SCL15*‐overexpressing lines, we show that SCL15 coordinates multiple protective mechanisms during seed maturation that underpin desiccation tolerance and longevity acquisition. These include ROS detoxification, DNA and protein repair, chlorophyll degradation, and the accumulation of LEA proteins, HSPs, and raffinose family oligosaccharides (RFOs). Our findings suggest that SCL15 acts as a central integrator of hormone signaling and stress‐response pathways, reinforcing antioxidant defenses during maturation to safeguard seed viability.

## 2 Methods

### 2.1 Plant Materials and Growth Conditions

The *A. thaliana* T‐DNA insertion line SALK_110871 (*scl15‐1*) was obtained from the Arabidopsis Biological Resource Centre (Ohio State University). Plants were grown under standard conditions as described previously (Gao et al. 2009, 2015). The overexpression lines using either the constitutively‐expressed *CaMV 35S* or seed/embryo‐specific *Napin* promoter are described in Gao et al. (2025).

### 2.2 Germination, Seed Viability, and Longevity Assays

Seeds that had been in dry storage for 7 days to 36 months in the dark under ambient room‐temperature conditions (relative humidity 45%–50% and 20°C–21°C) were surface‐sterilized and plated on half‐strength MS medium supplemented with 1% sucrose and 0.55% agar. After stratification for 3 days at 4°C, plates were transferred to 22°C under continuous light. Radicle protrusion was used as a marker for germination. Germination rates were recorded daily for 7 days and the average germination percentage was calculated.

Seed viability was evaluated using triphenyltetrazolium chloride (TTC) staining, which estimates cellular respiratory activity by detecting dehydrogenase enzyme function (Copeland and McDonald 2012). The assay was performed as described by Verma et al. (2013) with minor modifications. Briefly, approximately 100 seeds were scarified for 15 min at room temperature, thoroughly rinsed with distilled water, and then incubated in 1% (w/v) TTC solution in the dark at 30°C for 72 h. After incubation, seeds were washed to remove excess stain and examined under a Zeiss stereomicroscope for imaging.

Seed longevity was assessed for seeds in dry storage for 12 months and 36 months using an accelerated aging test (AAT) or controlled deterioration (CD) test as previously described (Nguyen et al. 2012, 2015), with seeds first equilibrated over saturated NaCl at room temperature for 5 days and then incubated at 42°C and 85% relative humidity for 5–7 days. Following CD, germination was assessed as described above. Seeds subjected to CD were also collected for RNA and biochemical analyses following 1‐h imbibition and immediate freezing in liquid nitrogen.

### 2.3 RNA Extraction and Reverse Transcription Quantitative PCR (RT–qPCR)

All genotypes were grown under identical environmental conditions, siliques were tagged to ensure equivalent developmental stages, and only uniformly light brown (mid‐stage) or fully mature dark brown (late‐stage) siliques were harvested. Siliques were collected at mid‐ and late‐stages of seed development: 12 and 18 days postanthesis (DPA) for wild type (WT) and *SCL15*‐overexpression lines, and 14 and 20 DPA for *scl15‐1* to account for delayed development (Gao et al. 2025). Total RNA was extracted and RT–qPCR was performed as previously described (Gao et al. 2015). *ACT2* and *EF‐1α* served as internal controls. Primer sequences are provided in Table S1.

### 2.4 RNA Sequencing and Data Analysis

Transcriptomic data from *A. thaliana* siliques were originally generated by Gao et al. (2025), who filtered out genes predominantly expressed in silique valves rather than seeds. Plant growth conditions were as described above. Total RNA was extracted from developing siliques of WT, *scl15‐1*, and Napin:SCL15 lines. Individual siliques were tagged and harvested as described above from Col and Napin:SCL15 lines at 16–18 DPA, whereas *scl15‐1* samples were collected at 18–20 DPA due to the approximately two‐day delay in silique development in this mutant (Gao et al. 2025).

The transcriptomic data were previously analyzed for Biological Process Gene Ontology (GO) categories related to seed dormancy using BiNGO (Gao et al. 2025). In the present study, the same dataset was reanalyzed using ShinyGO version 0.85.1 (Ge et al. 2020) and agriGO v2.0 (Tian et al. 2017). ShinyGO is a graphical tool for enrichment analysis based on annotation databases acquired from Ensembl and STRING‐db, which can link gene lists with molecular pathways and functional categories. Differentially expressed genes (DEGs) with a false discovery rate (FDR) cutoff value of 0.05 were imported for insight into their functional classifications (Ge et al. 2020).

### 2.5 Quantification of Hydrogen Peroxide, Superoxide, and Antioxidant Activity

Freshly harvested seeds or seeds stored dry for 90 days and then subjected to accelerated aging were used. Hydrogen peroxide content was quantified as described by Lariguet et al. (2013). Seeds were ground in 200 mM perchloric acid (1 mL/100 μg FW), centrifuged at 15,000*g* for 15 min at 4°C and then neutralized to pH 7.5. H_2_O_2_ was measured using a peroxidase‐based *O*‐dianisidine assay (560 nm), with results expressed in μmol g^−1^ weight.

Superoxide levels were assessed via nitroblue tetrazolium (NBT) reduction (Chaitanya and Naithani 1994). Seed homogenates (0.5 g) were extracted in phosphate buffer (0.2 M, pH 7.2) and 100 μL supernatant was incubated with 0.75 mM NBT, 25 mM sodium carbonate, 0.1 mM EDTA, and 13.3 mM L‐methionine at 30°C for 10 min. Absorbance was measured at 540 nm.

Total antioxidant capacity was assessed using the 2, 2‐diphenyl‐1‐picrylhydrazyl (DPPH) radical scavenging assay (Brand‐Williams et al. 1995). Seed extracts (2 mg in 1 mL methanol) were mixed with 0.06 mM DPPH, incubated for 30 min in the dark, and the absorbance was measured at 517 nm. Results were expressed as a percentage of radical scavenging activity.

### 2.6 Chlorophyll Content Measurement

Total chlorophyll content was quantified as described by Zhang et al. (2015). Freshly harvested seeds or seeds dry‐stored for 90 days were ground in liquid nitrogen and extracted in cold 80% acetone with 1 μM KOH. Extracts were centrifuged at 16,000*g* for 2 min at 4°C and absorbance was measured at 649 and 665 nm. Chlorophyll content was calculated as described by Strain et al. (1971).

### 2.7 Seed Oil, Fatty Acid Composition, and Protein Profile

Seed oil and fatty acid composition were analyzed using gas chromatography (Heydarian et al. 2016). Briefly, 100 mg seeds stored dry for 3 months were extracted with tripentadecanoin in hexane, derivatized using sodium methoxide, and analyzed on an Agilent 6890N GC system. Total oil contents of individual triglycerides were measured, and fatty acids were expressed as percentages of total methyl esters.

Protein concentration was determined using the Bradford assay (Bradford 1976) with a commercial kit (Cat. No. PA102, Tiangen Biotech). Briefly, 25 mg of seeds stored dry for 90 days were extracted in 250 μL buffer and 75 μL of the supernatant was analyzed at 595 nm. Protein profiles were examined using the Experion Pro260 automated electrophoresis system (Bio‐Rad) as described by Lyzenga et al. (2019). Triplicate protein samples (3000 ng/μL) from four plants were analyzed under reducing conditions. Protein fractions, including cruciferins and napins, were quantified as a percentage of total detected protein using Bio‐Rad Experion software (Figure S1).

### 2.8 Statistical Analysis

Statistical analyses of germination assays, ROS accumulation, antioxidant capacity, chlorophyll content, RT–qPCR data, protein content and oil content were performed using the Student's *t*‐test. Rosette leaf number and seed viability percentage were analyzed by one‐way analysis of variance (ANOVA). Data are presented as means ± SD from three independent experiments (*n* = 3), except for rosette leaf number (*n* = 15) and seed yield (*n* = 10).

## 3 Results

### 3.1 Global Transcriptomic Analysis Identifies Potential Functions for *SCL15* in Seed Longevity

Previous studies showed that loss of *SCL15* leads to markedly reduced expression of key seed maturation regulators, including *ABI3*, *CRA1* (Gao et al. 2015), and *ABI5* (Gao et al. 2025), particularly, during late seed development. Because ABI3, ABI5, and CRA1 play critical roles in seed longevity (Ooms et al. 1993; Sugliani et al. 2009; Zinsmeister et al. 2016), these observations suggest that SCL15 may influence longevity‐related processes during seed maturation. In the present study, altered *SCL15* transcript abundance also affected vegetative and reproductive growth, as well as seed development (Figure S2), further supporting a broader role for SCL15 in developmental regulation. To further investigate its biological functions, transcriptomic analyses were performed on maturing seeds of WT, the *scl15‐1* mutant, and a seed‐specific *SCL15* overexpression line (Napin:SCL15). These datasets, which previously demonstrated a role for SCL15 in dormancy release (Gao et al. 2025), were reanalyzed here using ShinyGO and agriGO to elucidate SCL15‐regulated pathways during seed maturation.

**FIGURE 1:**
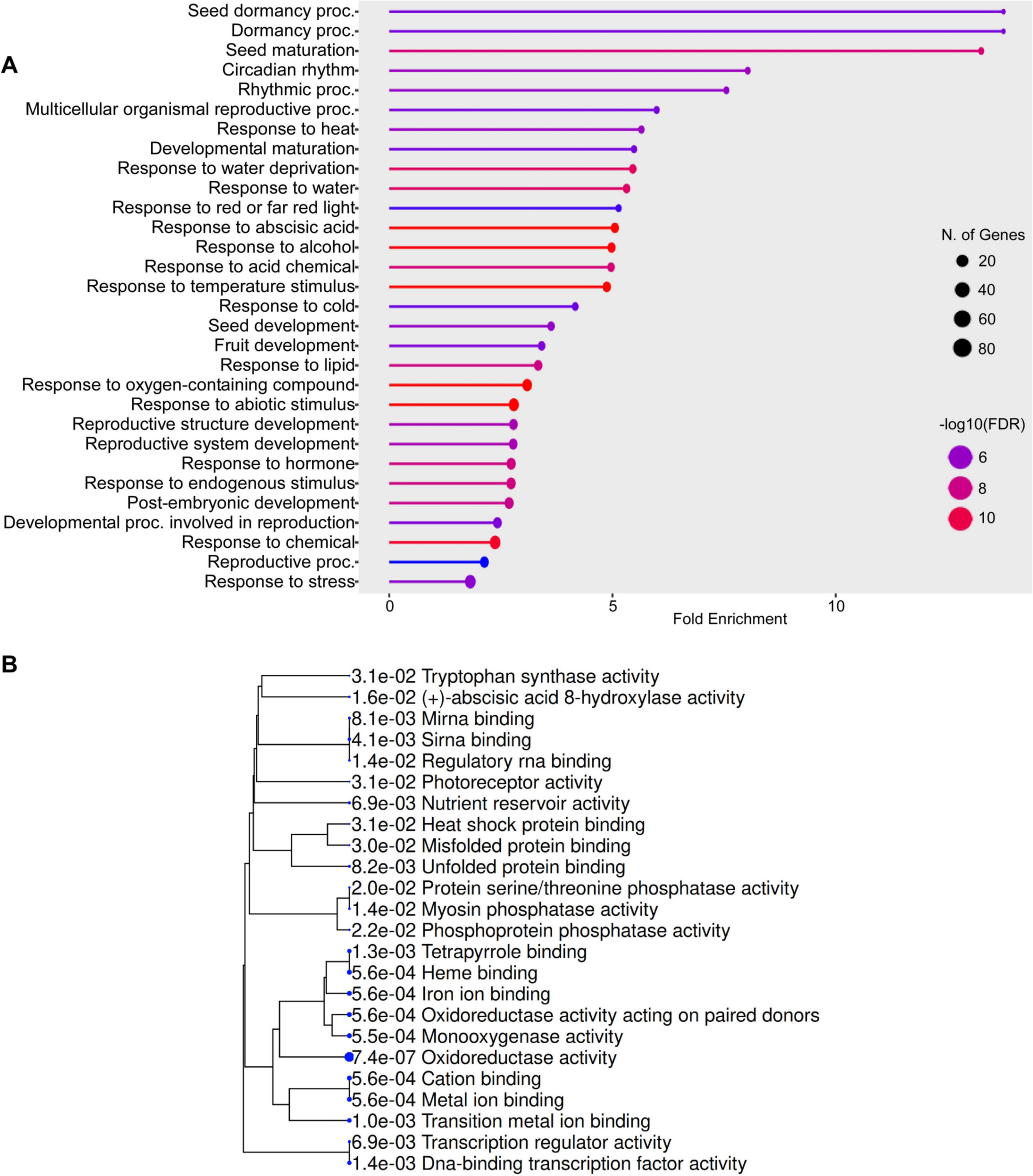
GO enrichment analysis of SCL15‐upregulated genes during seed maturation. ShinyGO was used to analyze 317 DEGs that were downregulated in *scl15‐1* and upregulated in Napin:SCL15, representing genes positively regulated by SCL15. (A) Enriched GO Biological Process terms. Circle size indicates the number of DEGs associated with each term and color intensity represents statistical significance (−log_10_ FDR). Fold enrichment corresponds to the proportion of DEGs in each term relative to the background gene set. (B) Enriched GO Molecular Function terms displayed as a hierarchical tree with adjusted *p*‐values shown next to each term. Larger dots indicate more significant enrichment with closely‐related terms that share more genes clustering together. Enriched categories highlight Biological Processes related to seed maturation, dormancy, stress responses, redox metabolism, protein protection and transcriptional regulation, while Molecular Function terms are associated primarily with redox regulation and cellular protection. ShinyGO analysis was performed using a false discovery rate (FDR) cutoff of 0.05 and the top 30 pathways are shown.

Among the 315 DEGs downregulated in *scl15‐1* and upregulated in Napin:SCL15, GO Biological Process enrichment revealed strong overrepresentation of terms related to seed dormancy, dormancy processes and seed maturation, which showed the highest fold enrichment among all categories (Figures 1A and S3). Additional enrichment of reproductive and postembryonic developmental processes indicates that SCL15 positively regulates maturation‐associated developmental programs. Notably, enriched categories included abscisic acid (ABA)–responsive processes and multiple stress‐related responses, including water deprivation, temperature stimuli (heat and cold), oxygen‐containing compounds, and general abiotic stress, all of which are central to the acquisition of desiccation tolerance and long‐term seed survival. GO molecular function analysis further revealed strong enrichment for functions related to redox regulation and cellular protection (Figure 1B), including oxidoreductase and monooxygenase activities, oxidoreductases acting on paired donors, as well as heme, tetrapyrrole, iron‐ion, and transition metal ion binding. These molecular functions are characteristic of enzymes involved in ROS metabolism, redox homeostasis, and oxidative damage control. In addition, as shown in Figure 1B, enrichment in HSP binding, misfolded protein binding, and unfolded protein binding suggests enhanced capacity for protein stabilization and repair, which is essential for maintaining macromolecular integrity during seed desiccation and storage.

**FIGURE 2:**
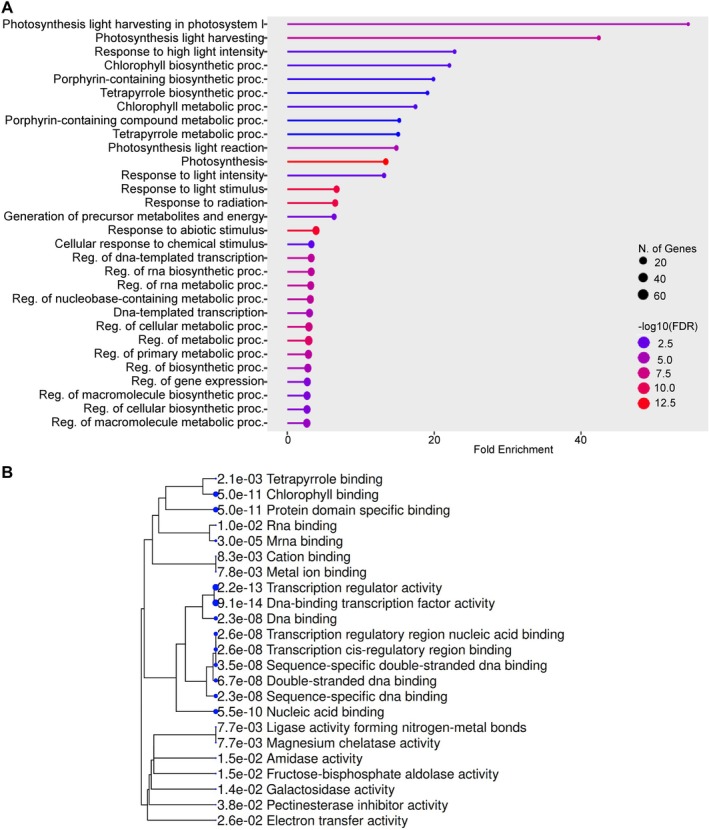
GO enrichment analysis of SCL15‐downregulated genes during seed maturation. ShinyGO was used to analyze 178 DEGs that were upregulated in *scl15‐1* and downregulated in Napin:SCL15, representing genes negatively regulated by SCL15. (A) Enriched GO biological process terms. (B) GO molecular function enrichment displayed as a hierarchical clustering dendrogram, with adjusted *p*‐values shown next to each term. Enriched categories predominantly relate to photosynthesis, chlorophyll and tetrapyrrole metabolism, transcriptional activity, and primary metabolic processes. ShinyGO analysis was performed using a false discovery rate (FDR) cutoff of 0.05 and the Top 30 pathways are shown.

Conversely, among the 178 DEGs upregulated in *scl15‐1* and downregulated in Napin:SCL15, GO Biological Process analysis revealed striking enrichment for photosynthesis‐related pathways, including photosynthesis light harvesting in photosystem I, photosynthesis light harvesting, photosynthesis light reactions, and chlorophyll biosynthetic and metabolic processes (Figures 2A and S4). Closely‐related categories, such as tetrapyrrole and porphyrin‐containing compound biosynthesis and metabolism, were also highly enriched. These processes are normally repressed during late seed maturation; their persistence in *scl15‐1* therefore indicates defective suppression of chloroplast‐associated metabolism. Enrichment of responses to highlight intensity, light stimulus and radiation further suggests continued photoresponsive signaling in the mutant. GO Molecular Function analysis supported these findings, with strong enrichment for chlorophyll and tetrapyrrole binding, magnesium chelatase activity and electron transfer activity, indicating persistence of redox‐active pathways capable of generating ROS (Figure 2B). Together, these results demonstrate that SCL15 positively regulates genes involved in H_2_O_2_ metabolism, antioxidant defense and cellular repair, while repressing chlorophyll biosynthesis and photosystem‐related genes during seed maturation.

### 3.2 Germination Assays and TTC Staining Validate a Positive Role for SCL15 in Seed Longevity

**FIGURE 3:**
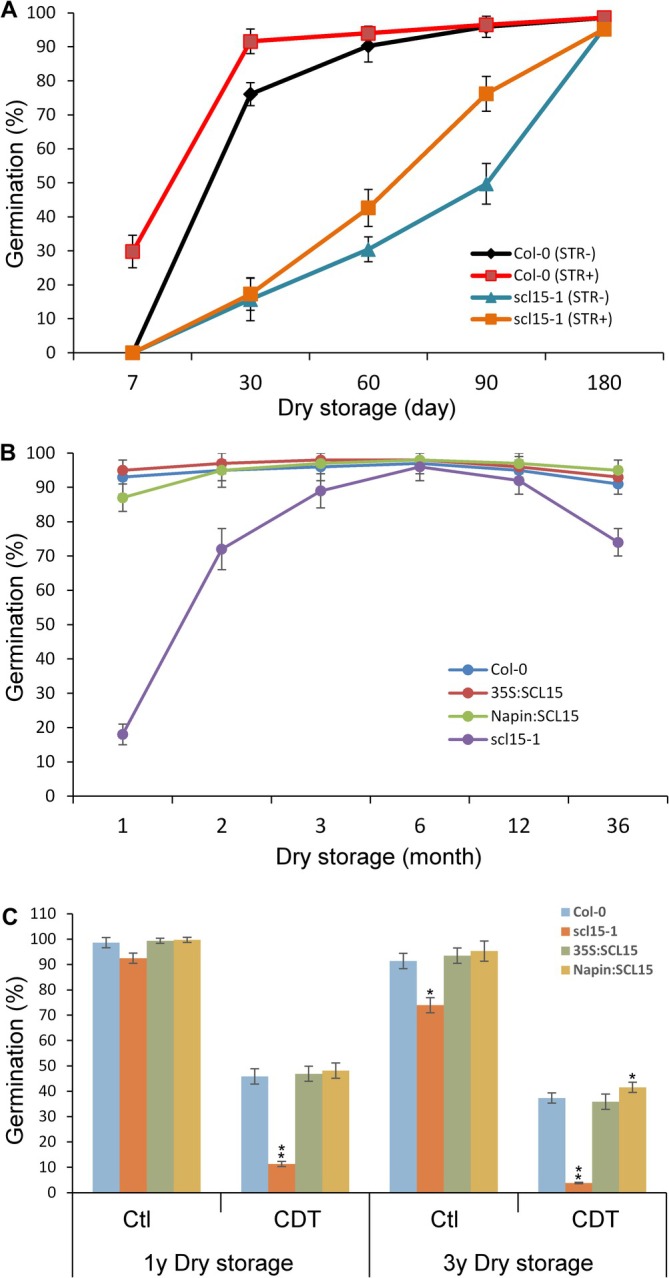
Germination behavior of *Arabidopsis thaliana* seeds during long‐term dry storage and after CDT treatment. (A) Germination comparison between WT and *scl15‐1* seeds with stratification (+STR) or without stratification (−STR) after dry storage for up to 6 months. For stratification, seeds were cold‐treated at 4°C for 3 days before sowing on ½ MS medium. Germination percentages represent means ± SD from three biological replicates. (B) Germination performance of WT, *scl15‐1*, and *SCL15*‐overexpressing lines (Napin:SCL15 and 35S:SCL15) following up to 3 years of dry storage at room temperature. (C) Germination rates of the same genotypes after 1 and 3 years of dry storage, with or without CDT. Seeds were subjected to CDT for 7 days, then germinated on ½ MS medium for 7 days. Germination values represent means ± SD from three biological replicates, each with five technical replicates of ~100 seeds per replicate. Asterisks indicate statistically significant differences compared to Col‐0 (*p < 0.05; **p < 0.01; Student's *t*‐test). Error bars represent ±SD (*n* = 3).

To assess whether SCL15 influences dormancy release and seed longevity, germination assays were performed using *scl15‐1*, WT, and *SCL15*‐overexpressing lines following dry storage for up to 3 years. Dormancy in WT was largely broken after 3 months of dry storage, with a germination rate exceeding 95%. In contrast, *scl15‐1* seeds exhibited less than 50% germination under the same conditions and required up to 6 months of dry storage for full dormancy release (Figure 3A). While stratification after 1 month of dry storage successfully overcame dormancy in WT, germination of *scl15‐1* remained below 20%, even with cold treatment (Figure 3A), consistent with enhanced dormancy in the mutant (Gao et al. 2025).

After six months of dry storage, when dormancy was considered fully broken, germination rates of *scl15‐1* exceeded 95%, comparable to WT and SCL15‐overexpressing lines, indicating that mutant seeds were viable at this stage. However, after 1 year and longer storage periods, *scl15‐1* seeds showed a more rapid decline in germination compared to WT and the *SCL15*‐overexpressing lines (Figure 3B), suggesting compromised seed storability in the mutant. These findings indicate that SCL15 negatively affects dormancy maintenance as previously demonstrated (Gao et al. 2025), while positively contributing to the maintenance of seed longevity during storage.

To further evaluate the role of SCL15 in seed longevity, a controlled deterioration treatment (CDT) was applied to seeds after 1 and 3 years of dry storage. Following 1 year of storage, CDT caused an 85% reduction in germination in *scl15‐1*, compared to a 50% reduction in WT. After 3 years, *scl15‐1* seeds exhibited an even greater reduction in germination (95%), whereas WT showed only a 60% reduction (Figure 3C). These results indicate that *scl15‐1* seeds are more sensitive to aging stress than WT and *SCL15*‐overexpressing lines.

To further evaluate seed viability after prolonged storage, TTC staining was performed on seeds stored for 7 and 9 years (Figure S5). The assay functioned as expected, with WT seeds stored for 1 year showing strong staining (positive control) and heat‐killed WT seeds showing little or no staining (negative control) (Figure S5A). TTC staining revealed similar overall staining patterns among WT, *scl15‐1*, Napin:SCL15, and 35S:SCL15 seeds after long‐term storage. Quantification of TTC‐positive seeds showed no statistically significant differences in viability among the genotypes after 7 or 9 years of storage, although the *scl15‐1* seed lot stored for nine years showed a slight reduction compared with WT (Figure S5B,C).

### 3.3 *SCL15* Regulates ROS Processing and Antioxidant Pathways During Seed Maturation

ROS levels during seed maturation are tightly controlled through a complex antioxidant network, including enzymes such as superoxide (O_2_•^−^) dismutase (SOD), which catalyzes the dismutation of superoxide radicals, and various hydrogen peroxide reductases like catalase (CAT), glutathione peroxidase (GPX), and peroxiredoxins (Prxs) (Mhamdi and Van Breusegem 2018; Wang et al. 2018). In this study, all identified genes encoding ROS‐detoxifying enzymes or components of antioxidant systems were downregulated in *scl15‐1* and upregulated in Napin:SCL15 seeds (Tables 1 and S2). These included *MSD2* (a mitochondrial manganese SOD) (Chen et al. 2022) and *GPX6* (Bela et al. 2015), both positively regulated by SCL15 based on transcriptomic analysis. Two genes encoding peroxiredoxins, *PER1* (also known as *1‐Cys Prx*) and *PER13* (Chen et al. 2016; Wang et al. 2022), were also positively regulated by SCL15 in maturing seeds.

**TABLE 1:** Representative seed longevity‐associated genes that were differentially expressed in *scl15‐1* and Napin:SCL15 seeds as identified by transcriptomic analysis.

Gene name	Locus identifier (AGI)	Expression (*scl15‐1* vs. WT)^d^	Expression (*Napin:SCL15* vs. WT)^d^	Molecular function in seed longevity
*Antioxidants*				
*MSD2* ^a^	AT3G56350	Down	Up	Encoding a superoxide dismutase catalyzing the conversion of superoxide radicals into O_2_ and H_2_O_2_ (Chen et al. 2022)
*GPX6*	AT4G11600	Down	Up	Encoding a glutathione peroxidase detoxifying H_2_O_2_ (Bela et al. 2015)
*PER1*	AT1G48130	Down	Up	Encoding a peroxide reductase detoxifying hydroxyl radicals and H_2_O_2_ (Wang et al. 2022)
*PER13*	AT1G77100	Down	Up	Encoding a peroxide reductase detoxifying hydroxyl radicals and H_2_O_2_ (Wang et al. 2018)
*GRXC1*	AT5G63030	Down	Up	Encoding an oxidoreductase involved in the protection against protein oxidative damage (Riondet et al. 2012)
*Chlorophyll metabolism and photosynthesis*				
*SGR2* ^b^	AT4G11910	Down	Up	Encoding STAY‐GREEN2 contributing to embryo‐specific degreening (Delmas et al. 2013)
*NYC1* ^b^	AT4G13250	Down	Up	Encoding NON‐YELLOW COLORING1 catalyzing the first step of chlorophyll catabolism (Delmas et al. 2013)
*Lhcb1.3* ^b^	AT1G29930	Up	Down	Encoding light‐harvesting complex (LHC) b1.3 primarily associated with Photosystem (PS) II (Pietrzykowska et al. 2014)
*Lhcb2.1* ^b^	AT2G05100	Up	Down	Encoding LHC b2.1 primarily associated with PSII (Pietrzykowska et al. 2014)
*PSAN* ^b^	AT5G64040	Up	Down	Encoding PSI subunit N (PSI‐N) (Rolo et al. 2024)
*Protein and DNA repair factors*				
*PIMT2* ^a^	AT5G50240	Down	Up	Encoding a protein repairing enzyme Protein l‐isoaspartyl Methyltransferase (Ogé et al. 2008)
*MSRB6*	AT4G04840	Down	Up	Encoding a protein repairing enzyme methionine sulfoxide reductase (Châtelain et al. 2013)
*PARP3*	AT5G22470	Down	Up	Encoding a base damage repairing enzyme poly (ADP‐ribose) polymerase (Boehler et al. 2011)
*AtWEE1*	AT1G02970	Up	Down	Encoding a conserved WEE1 homolog, a key player of DNA damage response (Pedroza‐García et al. 2022)
*CYCD3;2*	AT5G67260	Up	Down	Encoding D3‐type cyclin required for cell‐cycle‐regulated DNA damage repair (Collins et al. 2012)
*Protection proteins*				
*AtSTS*	AT4G01970	Down	Up	Encoding a stachyose synthase involved in seed vigor and longevity (Li et al. 2017)
*AGAL2*	AT5G08370	Up	Down	Encoding a α‐galactosidase involved in hydrolyzation of raffinose family oligosaccharides (Shabalin et al. 2002)
*AtEM6* ^a^	AT2G40170	Down	Up	Encoding EARLY METHIONINE‐LABELED 6 involved in desiccation tolerance (Manfre et al. 2009)
*LEA4‐2/LEA18* ^a^	AT2G35300	Down	Up	Encoding a group 4 LEA with chaperone‐like activity to protect enzymes in response to dehydration stress (Cuevas‐Velazquez et al. 2016; Rendón‐Luna et al. 2024).
*HSFA9*	AT5G54070	Down	Up	Encoding Heat Shock Transcription Factor A9, a key gene in desiccation tolerance and longevity (Zinsmeister et al. 2020; Wang et al. 2024)
*HSP17.4* ^a^	AT3G46230	Down	Up	Encoding heat shock protein 17.4, a molecular marker for oxidative stress (Zinsmeister et al. 2020)
*CRA* ^b^, ^c^	AT5G44120	Down	Up	Encoding Cruciferin A, a primary target for oxidation in seeds (Sano et al. 2016)
*CRC* ^b^	AT4G28520	Down	Up	Encoding Cruciferin C, a primary target for oxidation in seeds (Sano et al. 2016)
*ABA and auxin signaling factors*				
*CYP707A2*	AT2G29090	Down	Up	Encoding 8′‐hydroxylase for ABA catabolism, a positive regulator of seed longevity (He et al. 2014)
*ABI3* ^b^, ^c^	AT3G24650	Down	Up	Encoding ABA INSENSITIVE 3 involved in the positive regulation of seed longevity (Clerkx et al. 2004)
*ABI5* ^a^, ^b^	AT2G36270	Down	Up	Encoding ABA INSENSITIVE 5 involved in the positive regulation of seed longevity (Liu et al. 2013)
*DOG1* ^a^	AT5G45830	Down	Up	Encoding DELAY OF GERMINATION 1 involved in the positive regulation of seed longevity (Bentsink et al. 2006)
*IAA27*	AT4G29080	Up	Down	Encoding AUX/IAA PROTEIN 27 involved in regulating longevity by repression of the *HSFA9* (Carranco et al. 2010)

Abbreviations: AGI, arabidopsis genome initiative; Down, downregulation.

^a^ Validated previously by RT‐qPCR analysis (Gao et al. 2025).

^b^ Validate by real‐time RT‐qPCR analysis in the present study.

^c^ Validated previously by RT‐qPCR analysis (Gao et al. 2015).

^d^ Fold change > 1.5 (*p* < 0.05); Up, upregulation, fold change > 1.5 (*p* < 0.05).

Beyond genes encoding classical ROS‐processing systems, additional antioxidant‐related pathways were impacted by SCL15. Genes encoding members of the thioredoxin superfamily, such as *GRXC1* and *GRXS3 (*Riondet et al. 2012), were significantly downregulated in *scl15‐1* and upregulated in Napin:SCL15. Notably, *GRXC1* is seed‐specific, while *GRXS3* is expressed in the endosperm and seed coat (Arabidopsis eFP Browser). Genes encoding two glyoxalase I enzymes, *GLYI4* and *GLYI8*, which detoxify reactive carbonyl species (Schmitz et al. 2018), were also upregulated by SCL15. Of these, *GLYI8* is the only *GLYI* gene highly expressed in seeds. Furthermore, several genes encoding glutathione transferases from the tau class (GSTU), including *GSTU3* and its homolog *GSTU4* (Dixon et al. 2002), were downregulated in *scl15‐1* and upregulated in Napin:SCL15, reinforcing the role of SCL15 in enhancing antioxidant capacity during seed maturation.

**FIGURE 4:**
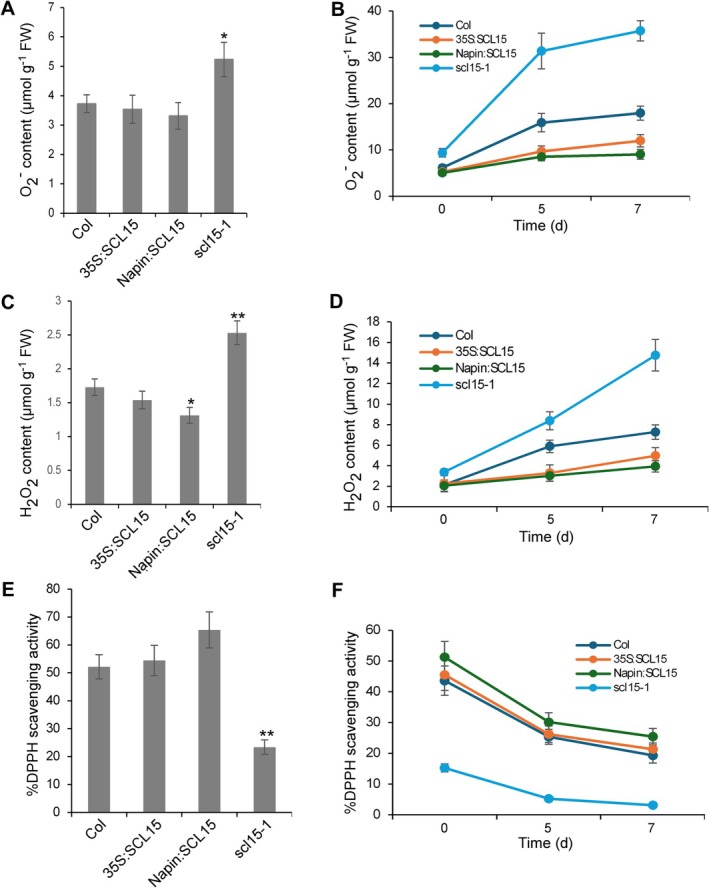
Measurement of ROS accumulation and antioxidant capacity in WT, *scl15‐1* mutant, and *SCL15*‐overexpression lines (35S:SCL15 and Napin:SCL15). Freshly harvested seeds (A, C, E) and dry‐stored seeds subjected to accelerated aging (B, D, F) were analyzed. (A) O_2_•^−^ levels in freshly harvested seeds. (B) O_2_•^−^ accumulation after 5 or 7 days of accelerated aging following 90 days of dry storage. (C) H_2_O_2_ levels in freshly harvested seeds. (D) H_2_O_2_ accumulation during accelerated aging. (E) Total antioxidant activity in freshly harvested seeds. (F) Total antioxidant activity after accelerated aging. Asterisks indicate statistically significant differences compared to wild‐type Col‐0 (*p < 0.05; **p < 0.01; Student's *t*‐test). Error bars represent ±SD (*n* = 3).

To validate the functional impact of SCL15 on ROS levels and antioxidant defense, O_2_•^−^, H_2_O_2_ content and total antioxidant activity in freshly harvested and CDT‐treated seeds were measured. Compared to WT and *SCL15*‐overexpressing lines, *scl15‐1* seeds showed significantly higher levels of both superoxide and hydrogen peroxide at harvest (Figure 4A,C). After 7 days of accelerated aging, ROS accumulation was further elevated in all genotypes, but was approximately two‐fold higher in *scl15‐1* than in WT (Figure 4B,D).

To assess antioxidant capacity, 2,2‐diphenyl‐1‐picrylhydrazyl (DPPH) assays were conducted. Total antioxidant activity was markedly lower in *scl15‐1* seeds compared to WT and *SCL15*‐overexpression lines, both in freshly harvested and aged seeds (Figure 4E,F). These physiological results are consistent with transcriptomic data and support a role for SCL15 in promoting ROS detoxification and antioxidant defense, thereby contributing to the acquisition of seed longevity.

### 3.4 SCL15 Regulates Chlorophyll Degradation and Photosystem‐Associated Gene Expression

**FIGURE 5:**
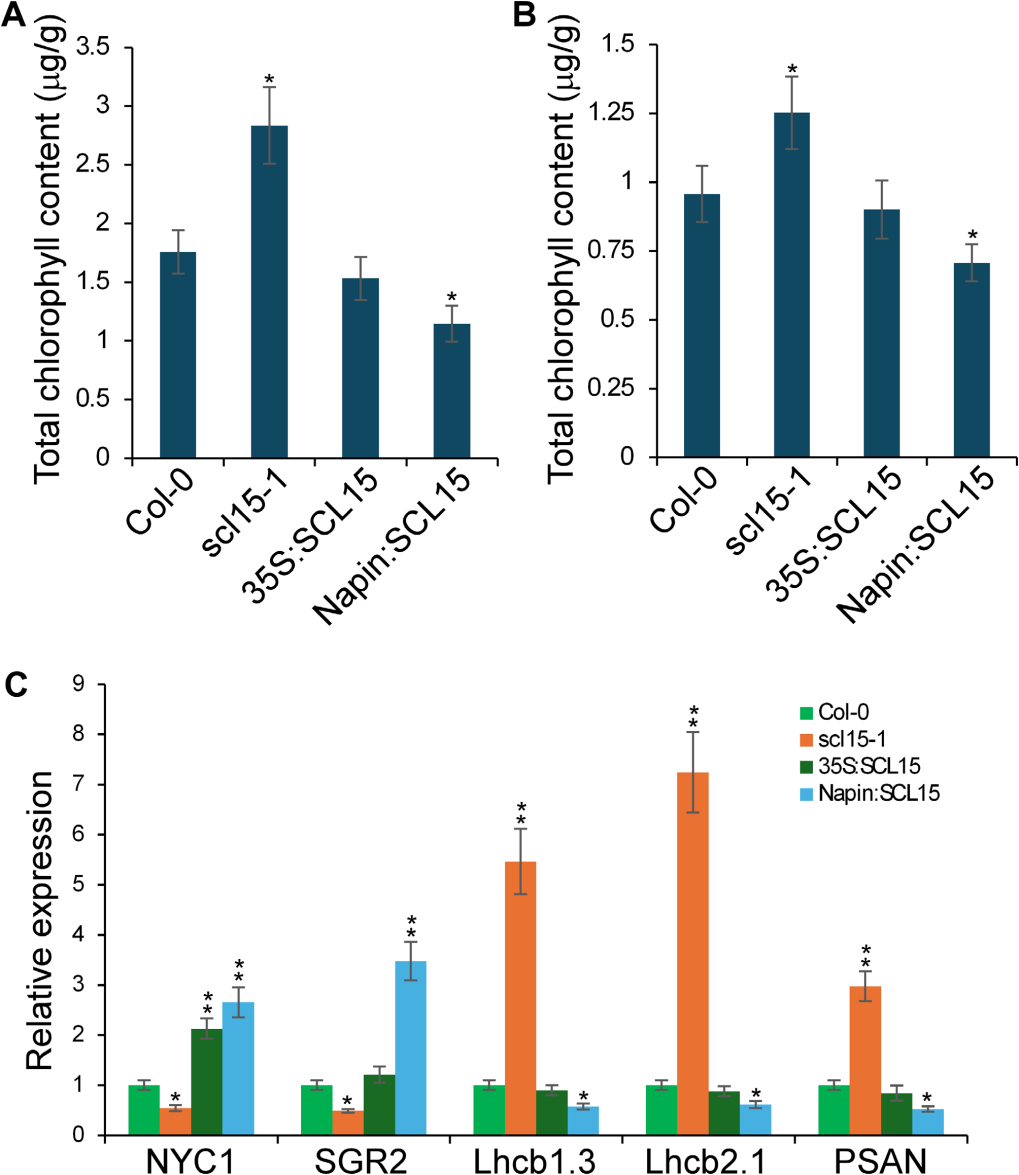
Chlorophyll content and photosystem‐associated gene expression are altered in *scl15‐1* seeds. (A) Chlorophyll accumulation in freshly harvested seeds. (B) Chlorophyll accumulation in after‐ripened seeds following 90 days of dry storage. (C) RT‐qPCR analysis showing the relative expression levels of *NYC1* and *SGR2* (chlorophyll degradation genes), *PSAN* (photosystem I subunit), and *CAB1/Lhcb1.3* and *Lhcb2.1* (photosystem II light‐harvesting complex genes) in maturing seeds of WT, *scl15‐1* mutant and *SCL15*‐overexpression lines (35S:SCL15 and Napin:SCL15). Asterisks indicate statistically significant differences relative to Col‐0 (*p* < 0.05; *p* < 0.01; Student's *t*‐test). Error bars represent ±SD (*n* = 3).

During the seed maturation phase, chlorophyll is actively degraded through a well‐coordinated pathway involving enzymes such as NON‐YELLOW COLORING1 (NYC1) and STAY‐GREEN2 (SGR2). This process, known as “seed degreening,” is essential for seed quality and longevity. To determine whether SCL15 influences chlorophyll breakdown, total chlorophyll content was quantified in both freshly harvested and after‐ripened seeds. This analysis showed elevated chlorophyll levels in *scl15‐1* mutant seeds and reduced levels in Napin:SCL15 seeds relative to WT (Figure 5A,B). This pattern was consistent with the transcriptomic data, which revealed that *NYC1* and *SGR2* were significantly downregulated in *scl15‐1* and upregulated in *Napin:SCL15* (Figure 5C; Tables 1 and S2).

Additionally, transcriptomic analysis identified sustained expression of genes encoding photosystem subunits in *scl15‐1* seeds, including *PSAN* (a PSI‐associated subunit) and *Lhcb1* and *Lhcb2* (light‐harvesting chlorophyll a/b‐binding proteins PSII components) (Figure 5C; Tables 1 and S2). These transcripts were largely absent in WT seeds at maturity, indicating that the failure to properly degrade photosynthetic components in *scl15‐1* seeds may contribute to aberrant chlorophyll retention and impaired seed maturation.

### 3.5 SCL15 Affects Protein and DNA Repair Systems in Maturing Seeds

Oxidative damage to proteins, such as the formation of methionine sulfoxide (MetO) via methionine oxidation, can be reversed by methionine sulfoxide reductases (MSRs) (Stadtman 2006). Among the MSR gene family, expression of *MSRB5* and *MSRB6* (Cai et al. 2023) was affected by SCL15 abundance, with the embryo‐enriched *MSRB6* significantly downregulated in *scl15‐1* and upregulated in Napin:SCL15 lines. Additionally, proteins containing isoaspartyl (isoAsp) residues, which arise from spontaneous deamidation or isomerization and can compromise protein structure and function, are repaired by protein l‐isoaspartyl *O*‐methyltransferase (PIMT). This repair activity is critical for maintaining seed vigor and longevity in orthodox seeds (Ogé et al. 2008). The *A. thaliana* genome has two genes encoding PIMT isoforms: *PIMT1*, which is broadly expressed, and *PIMT2*, which is predominantly expressed in the seed. Transcriptomic analysis revealed strong downregulation of *PIMT2* in *scl15‐1* and upregulation in Napin:SCL15 seeds (Table 1), suggesting a role for SCL15 in protecting protein integrity during maturation.

SCL15 also regulates genes involved in DNA repair. Expression of *PARP3*, which encodes a poly(ADP‐ribose) polymerase that positively contributes to seed longevity (Rissel et al. 2014), was significantly reduced in *scl15‐1* and upregulated in Napin:SCL15 seeds (Table 1). In contrast, *AtWEE1*, which encodes a key cell cycle checkpoint regulator (Pedroza‐García et al. 2022), was upregulated in *scl15‐1* and suppressed in Napin:SCL15 seeds. SCL15 also negatively regulated the expression of several genes encoding cyclins (CYCs) and cyclin‐dependent kinases (CDKs), including *CDKB2;2*, *CYCD3;2*, and *CYCP4;1*, all of which showed elevated transcript levels in *scl15‐1* and reduced expression in Napin:SCL15. Together, these data support a role for SCL15 in maintaining seed viability through the regulation of both protein and DNA repair pathways during seed maturation.

### 3.6 SCL15 Influences Seed Protection Systems During Maturation

*A. thaliana* seeds accumulate several RFOs, including raffinose, stachyose, and verbascose, which contribute to seed desiccation tolerance and longevity. The seed‐specific gene *RAFFINOSE SYNTHASE 4* (*AtRS4*/, *AtSTS*) encodes an enzyme with stachyose synthase activity and limited raffinose synthase activity (Gangl and Tenhaken 2016), and has been positively associated with seed vigor and longevity (Li et al. 2017). In this study, *AtSTS* expression was downregulated in *scl15‐1* and upregulated in Napin:SCL15 seeds. Conversely, expression of *α‐GALACTOSIDASE 2* (*AGAL2*) (Shabalin et al. 2002), which encodes a major RFO catabolic enzyme, was elevated in *scl15‐1* and repressed in Napin:SCL15, indicating that SCL15 positively regulates RFO accumulation in maturing seeds.

SCL15 also regulated the expression of genes encoding LEA proteins, which play key roles in glassy‐state formation, desiccation tolerance and abiotic stress protection. Of the 34 LEA genes known to be seed‐specific or highly expressed during seed maturation (Hundertmark and Hincha 2008), 26 were differentially expressed in an SCL15‐dependent manner; all were downregulated in *scl15‐1* and upregulated in Napin:SCL15 (Tables 1 and S2). These include: (1) Group 1 LEA genes *AtEM1* and *AtEM6*, which are direct targets of ABI5 and regulate desiccation timing and water loss during seed maturation (Manfre et al. 2009); (2) Group 4 LEA genes *LEA4‐1*, *LEA4‐2* and *LEA4‐5*, known for chaperone‐like functions in protecting enzyme activity under dehydration (Cuevas‐Velazquez et al. 2016; Rendón‐Luna et al. 2024); and (3) multiple genes encoding dehydrins.

Genes encoding HSPs, another critical component of seed protection, are transcriptionally regulated by heat shock transcription factors (HSFs) (Wehmeyer and Vierling 2000). Among these, HSFA1 members serve as master regulators of the heat shock response (Liu et al. 2011), while the seed‐specific HSFA9 regulates HSP gene expression through interactions with ABA and auxin signaling pathways during seed maturation (Huang et al. 2016; Wang et al. 2024). In this study, both *HSFA1E* and *HSFA9* were downregulated in *scl15‐1* and upregulated in Napin:SCL15 seeds, suggesting that SCL15 positively influences their expression. Consistent with changes in HSF expression, several HSP genes were also regulated by SCL15. For instance, *HSP17.4*, *HSP17.6*, and *HSP101*, all known targets of HSFA9 and ABI3 (Zinsmeister et al. 2020), were repressed in *scl15‐1* and activated in Napin:SCL15 seeds (Table 1). These findings suggest that SCL15 plays a critical role in activating seed protection pathways to enhance seed longevity and resilience during maturation.

### 3.7 Altered Seed Storage Reserve Accumulation in *scl15‐1* Seeds

**FIGURE 6:**
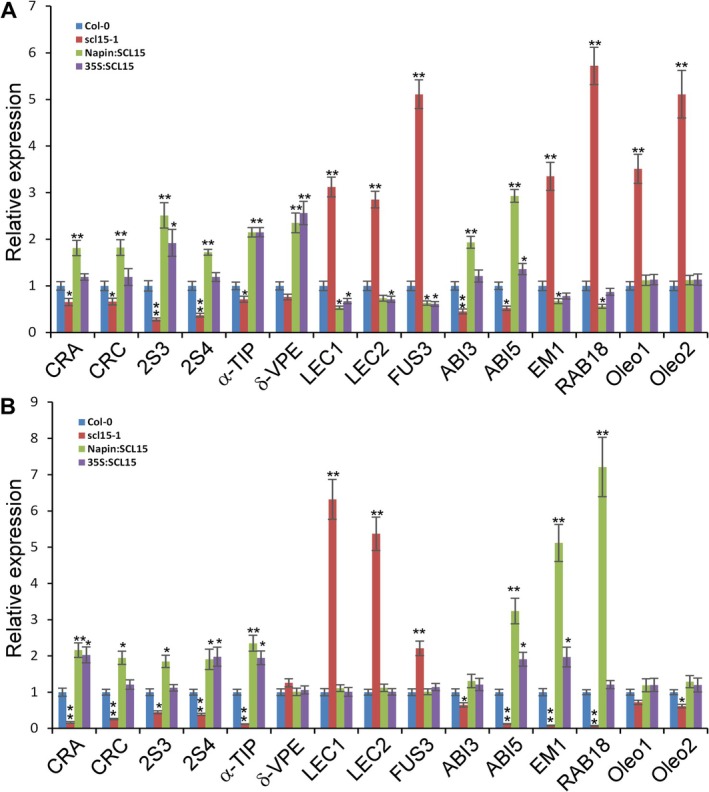
Expression of major seed maturation genes in *scl15‐1*, *SCL15*‐overexpression lines (Napin:SCL15 and 35S:SCL15) and WT during seed development. (A) Mid‐stages of seed development. (B) Late‐stages of seed development. Expression levels in the control WT were designated as one‐fold. Asterisks indicate a statistically significant difference between wild‐type Col‐0 and mutant *scl15‐1*, *SCL15*‐overexpression lines (*p < 0.05; **p < 0.01; Student's *t*‐test).

Seed storage proteins (SSPs) are synthesized during the seed‐filling phase under the control of key transcriptional regulators such as ABI3. The *A. thaliana* genome has three genes encoding 12S globulins, *CRUCIFERIN A* (*CRA*), *CRUCIFERIN B* (*CRB*), and *CRUCIFERIN C* (*CRC*), all of which were found to be downregulated in *scl15‐1* and upregulated in Napin:SCL15 lines based on transcriptomic analysis (Table 1). To further examine the role of SCL15 in SSP regulation during seed maturation, we conducted RT‐qPCR analysis of SSP genes, including those encoding for 2S albumins/napins and 12S globulins, proteins involved in SSP processing and trafficking (*α‐TIP* and *δ‐VPE*), and key seed maturation regulators (*LEC1*, *LEC2*, *ABI3*, *ABI5*, and *FUS3*) in *scl15‐1*, WT, and *SCL15*‐overexpression lines (Figure 6). Most SSP‐related genes exhibited reduced expression in *scl15‐1* compared to WT, especially during the late stages of embryo development (Figure 6B), whereas expression was restored or enhanced in the overexpression lines.

**FIGURE 7:**
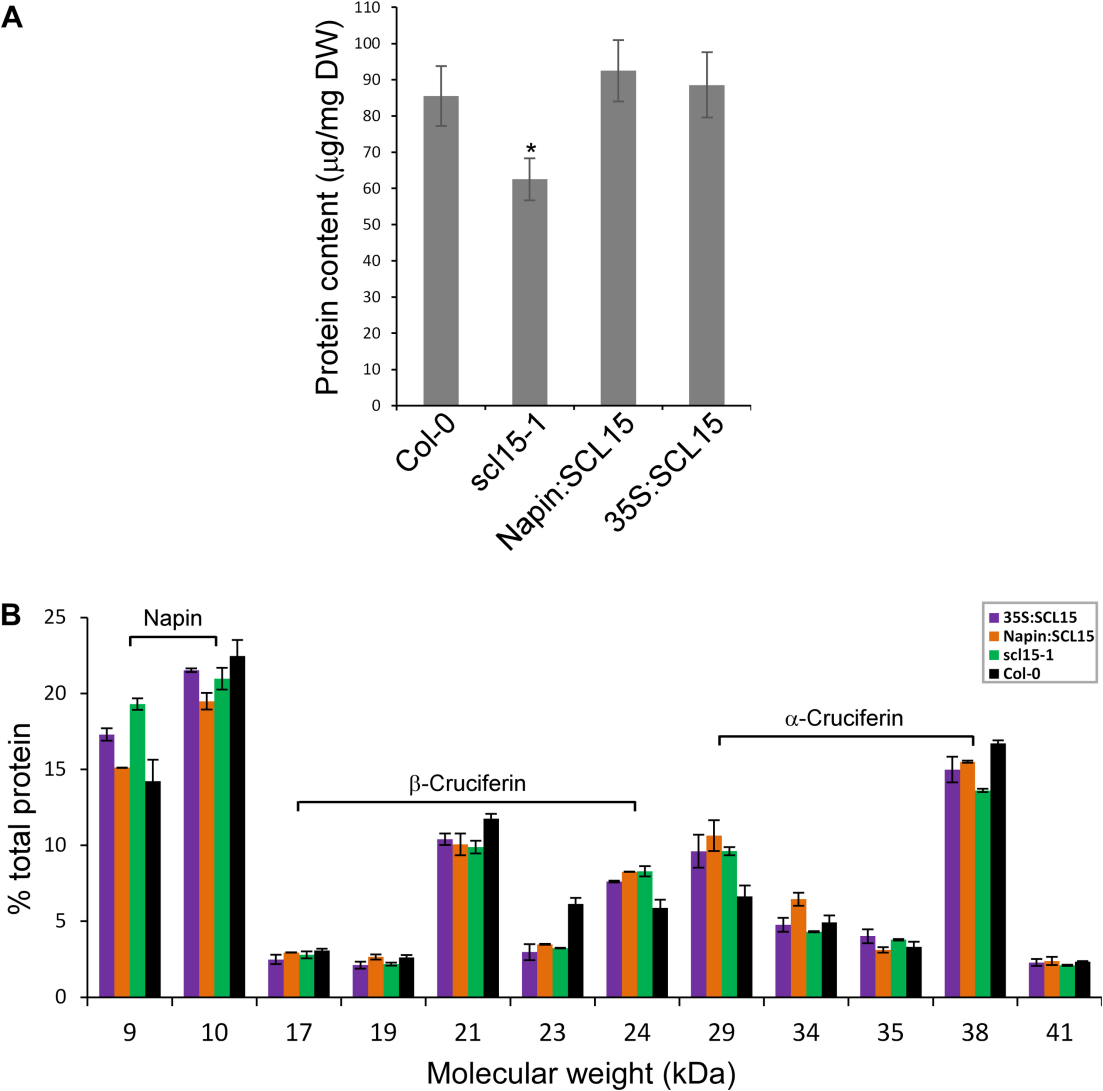
Alteration of SSP accumulation with *SCL15* transcript abundance in *Arabidopsis thaliana* seeds. (A) Seed protein content in WT, *scl15‐1* mutant and *SCL15*‐overexpression lines (Napin:SCL15 and 35S:SCL15). Data represent the mean ± SD of measurements of individual plants (*n* = 10). Asterisk indicates a statistically significant difference between Col‐0 and *scl15‐1* or *SCL15*‐overexpression lines (*p < 0.05; Student's *t*‐test). (B) Quantification of the electrochromatogram peak area as calculated by Experion software. Data represent the mean ± SD of triplicate measurements from each of three biological replicates.

Consistent with gene expression data, total protein content was significantly reduced in *scl15‐1* seeds compared to WT (Figure 7A). Protein profiling by microfluidic electrophoresis (Lyzenga et al. 2019) further revealed notable changes in SSP composition (Figure 7B). Under reducing conditions, the small and large subunits of 2S napins were detected at ~9 and ~10 kDa, while the α‐ and β‐chains of 12S cruciferins were detected at ~29–38 and ~17–24 kDa, respectively (Withana‐Gamage et al. 2013). Notably, the major α‐chain (38 kDa) and β‐chain (21 kDa) components of 12S cruciferins were markedly reduced in *scl15‐1* seeds compared to WT and *SCL15*‐overexpressing lines. These findings indicate that SCL15 is required for the proper accumulation of SSPs, particularly 12S cruciferins, during seed maturation.

**FIGURE 8:**
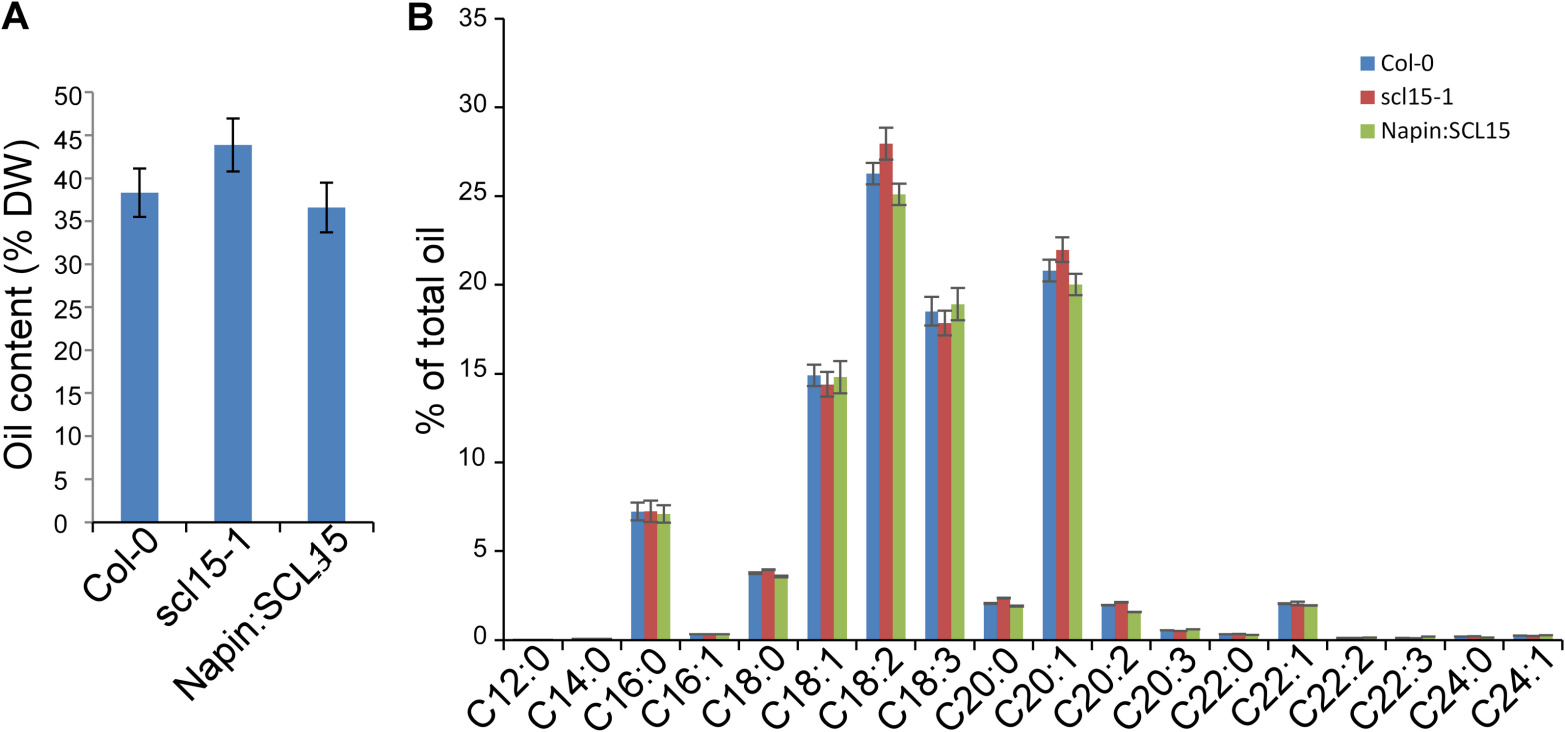
Regulation of seed oil accumulation by SCL15 in *Arabidopsis thaliana*. (A) Seed oil content of the WT, *scl15‐1* mutant and the *SCL15*‐overexpressing line Napin:SCL15. (B) Fatty acid compositions of the WT, *scl15‐1* and Napin:SCL15. Values are means ± SD (*n* = 3).

Whether SCL15 influences lipid reserves was assessed since seed oil content and composition are also linked to seed longevity in oilseed species such as *A. thaliana* , largely due to the susceptibility of unsaturated fatty acids to oxidative degradation (Silva et al. 2023). Interestingly, *scl15‐1* seeds exhibited a moderate but consistent increase (~14%) in total seed oil content compared to WT (Figure 8A). Fatty acid composition was largely unchanged, with the exception of linoleic acid (C18:2) and eicosenoic acid (C20:1), both of which were elevated in *scl15‐1* seeds relative to WT and the *SCL15*‐overexpression lines (Figure 8B). These results collectively demonstrate that SCL15 plays a role in regulating the accumulation of seed storage reserves, both protein and lipid, during maturation, and that this regulation may contribute to seed quality and longevity.

## 4 Discussion

Through integrated transcriptomic, physiological and biochemical analyses, SCL15 (AtHAM4) was identified as a positive regulator of seed longevity. SCL15 is a GRAS transcription factor in the HAM family, previously linked to WUSCHEL‐mediated stem cell regulation (Engstrom et al. 2011; Zhou et al. 2015), but unlike AtHAM1–AtHAM3, it is not regulated by miR171, suggesting functional divergence (Tian et al. 2004; Liu and Widmer 2014). Its strong expression in vasculature and developing seeds, particularly in companion cells at the chalaza and placentochalaza, key sites for nutrient unloading (Figure S6) (Gao et al. 2015; Zhou et al. 2015), supports a specialized role in maturation. Earlier work showed that SCL15 represses embryonic gene expression in seedlings through HDA19 but promotes maturation, as *scl15‐1* mutants exhibit reduced expression of *ABI3* and *CRA1* (Gao et al. 2015). More recently, SCL15 was reported to negatively regulate seed dormancy by modulating endosperm wall remodeling (Gao et al. 2025). Our findings build on this dual role, revealing that SCL15 enhances desiccation tolerance and longevity by strengthening protective and repair systems, particularly, those mitigating oxidative stress during late seed development. Together, these results establish SCL15 as a central regulator balancing seed dormancy, maturation, and longevity in *A. thaliana* .

Although ABA levels remain relatively stable during seed aging, ABA signaling components, particularly ABI3, ABI5 and HSFA9, are crucial for establishing seed viability during maturation and act in concert with auxin signaling to regulate protective responses (Carranco et al. 2010; Sano et al. 2016; Pellizzaro et al. 2020; Wang et al. 2024). Here, SCL15 was shown to promote the biosynthesis of protective molecules such as RFOs, LEA proteins, small HSPs and cruciferin‐type SSPs, which are essential for forming a cytoplasmic glassy matrix. This matrix helps arrest metabolism and preserve macromolecular integrity during desiccation (Sano et al. 2016). RFOs mitigate oxidative damage and HSPs function as chaperones that stabilize proteins and membranes under stress (Verdier et al. 2013; Lima et al. 2017; Ohama et al. 2017). Seed storage reserves also affect longevity; SSPs, especially 12S globulins, act as antioxidants buffering ROS during storage (Nguyen et al. 2015). In *scl15‐1* mutants, reduced expression of three 12S globulin genes coincided with increased oil content and linoleic acid levels, both of which are negatively associated with seed vigor (Nguyen et al. 2015; Silva et al. 2023).

HSFA9, which is activated by ABA signaling via ABI3 and repressed by the auxin‐responsive factor IAA27 (Carranco et al. 2010), coordinates the expression of HSPs that both respond to ROS and enhance antioxidant capacity (Ul Haq et al. 2019). The coordinated upregulation of RFO biosynthetic genes (*AtRS4*, *AtSTS*), LEAs (*AtEM1*, *AtEM6*, *LEA4s*), small HSPs (*HSP17.4*, *HSP17.6*), and *HSFA9* in *SCL15*‐overexpression lines, and their downregulation in the *scl15‐1*, underscore a central role for SCL15 in enhancing desiccation tolerance and longevity acquisition. ABI5‐mediated LEA expression and ABI3‐driven HSFA9 activation (Carranco et al. 2010; Zinsmeister et al. 2020), together with repression of *IAA27* by SCL15, suggest that SCL15 modulates longevity through coordinated ABA–auxin crosstalk converging on the HSFA9–HSP module.

Efficient chlorophyll degradation during late seed development is critical to prevent photooxidative damage. The altered expression of *NYC1* and *SGR2* in *scl15‐1* and overexpression lines implicates SCL15 in seed degreening. SGR2 not only facilitates chlorophyll breakdown but also dismantles photosynthetic complexes (Shimoda et al. 2016). Both *SGR2* and *NYC1* are regulated by ABI3 and ABI5, which are positively regulated by SCL15, reinforcing its role in promoting chlorophyll degradation and seed quality (Sakuraba et al. 2012; Delmas et al. 2013; Zinsmeister et al. 2016). Beyond protective measures, seeds activate repair mechanisms to mitigate macromolecular damage accumulated during maturation and early storage. SCL15 positively regulates genes involved in protein and DNA repair. Oxidized methionine residues are reversed by MSRs, which improves seed stress tolerance and viability (Châtelain et al. 2013; Hazra et al. 2022; Cai et al. 2023), while PIMTs repair isoAsp residues in critical transcription factors, such as ABI3 and ABI5, and are essential for desiccation tolerance (Ogé et al. 2008; Verma et al. 2013; Kamble and Majee 2022). Both MSR and PIMT genes were upregulated by SCL15, suggesting a coordinated reinforcement of protein repair capacity. Desiccation also leads to DNA damage, primarily from ROS‐induced strand breaks (Bray and West 2005; Zhou et al. 2020). DNA damage response (DDR) pathways, which activate repair machinery and cell cycle checkpoints, are therefore critical for seed viability (Nisa et al. 2019). In *scl15‐1* seeds, downregulation of *PARP3*, a core component of the DDR and a positive regulator of seed longevity (Boehler et al. 2011; Rissel et al. 2014), together with upregulation of *WEE1* and several *CDK/CYC* genes, indicative of G2/M checkpoint activation and homologous recombination repair (Weimer et al. 2016; Pedroza‐García et al. 2022), suggests compromised DDR homeostasis in the absence of SCL15.

Longevity phenotypes in this study were assessed using both natural aging and CDT, which accelerates oxidative and moisture‐dependent damage to provide a rapid and reproducible proxy for storage performance. While CDT does not fully recapitulate all biochemical processes occurring during long‐term dry storage, it reliably reflects intrinsic seed robustness and correlates with natural aging across genotypes (Bailly et al. 2008; Rehmani et al. 2022). The consistent phenotypes across CDT and dry storage therefore support a primary role for SCL15 in longevity acquisition during maturation rather than in downstream deterioration processes during storage. TTC staining of seeds after prolonged storage, which detects cellular respiratory dehydrogenase activity as an indicator of metabolic viability (Copeland and McDonald 2012), revealed broadly similar staining patterns among WT, *scl15‐1*, and the *SCL15*‐overexpression lines. These results indicate that a substantial proportion of seeds from all genotypes retained detectable metabolic activity even after extended storage. When considered together with the germination and CDT assays, the TTC results indicate that the detected phenotypic differences primarily reflect variation in seed vigor and storage robustness rather than loss of cellular viability. Such distinctions are consistent with previous studies showing that tetrazolium‐based viability assays may detect metabolically‐active embryos that nevertheless exhibit reduced germination performance due to progressive physiological deterioration during storage (Delouche and Baskin 1973; McDonald 1999; Copeland and McDonald 2001). Importantly, the primary objective of this study was to understand how SCL15 contributes to the acquisition of seed longevity during maturation. The transcriptomic, physiological, and biochemical analyses in this study consistently indicate that SCL15 regulates multiple maturation‐associated processes, including accumulation of protective molecules, ROS homeostasis, chlorophyll degradation and macromolecular repair systems. These pathways are known determinants of the intrinsic longevity potential established during seed development. Therefore, while deterioration processes occurring during long‐term storage undoubtedly influence final germination outcomes, the evidence presented here supports a model in which SCL15 acts primarily during seed maturation to establish the protective and metabolic state that ultimately determines seed longevity.

Dormancy release in after‐ripened *scl15‐1* seeds was delayed, and these seeds displayed markedly reduced sensitivity to cold stratification compared with WT. While WT dormancy was fully released after 1 month of storage plus cold treatment, *scl15‐1* germination remained below 20%. Cold stratification promotes germination by altering hormone balances, lowering ABA, enhancing GA, mobilizing reserves and altering embryo and endosperm structure (Bewley 1997; Bailly et al. 2008; Chen et al. 2015). Mild ROS accumulation also functions as a signal to repress ABA, stimulate GA synthesis and weaken the endosperm (Bailly 2019; Jhanji et al. 2024), whereas excessive ROS damages macromolecules and reduces seed vigor (Bailly et al. 2008; Anand et al. 2019). In *scl15‐1*, elevated ROS levels and reduced expression of ROS‐processing and repair genes likely disrupt this balance, impairing both ROS‐mediated signaling and hormone reprogramming during stratification. Thus, SCL15 is suggested to integrate ROS homeostasis with hormonal regulation to coordinate dormancy release and seed longevity.

**FIGURE 9:**
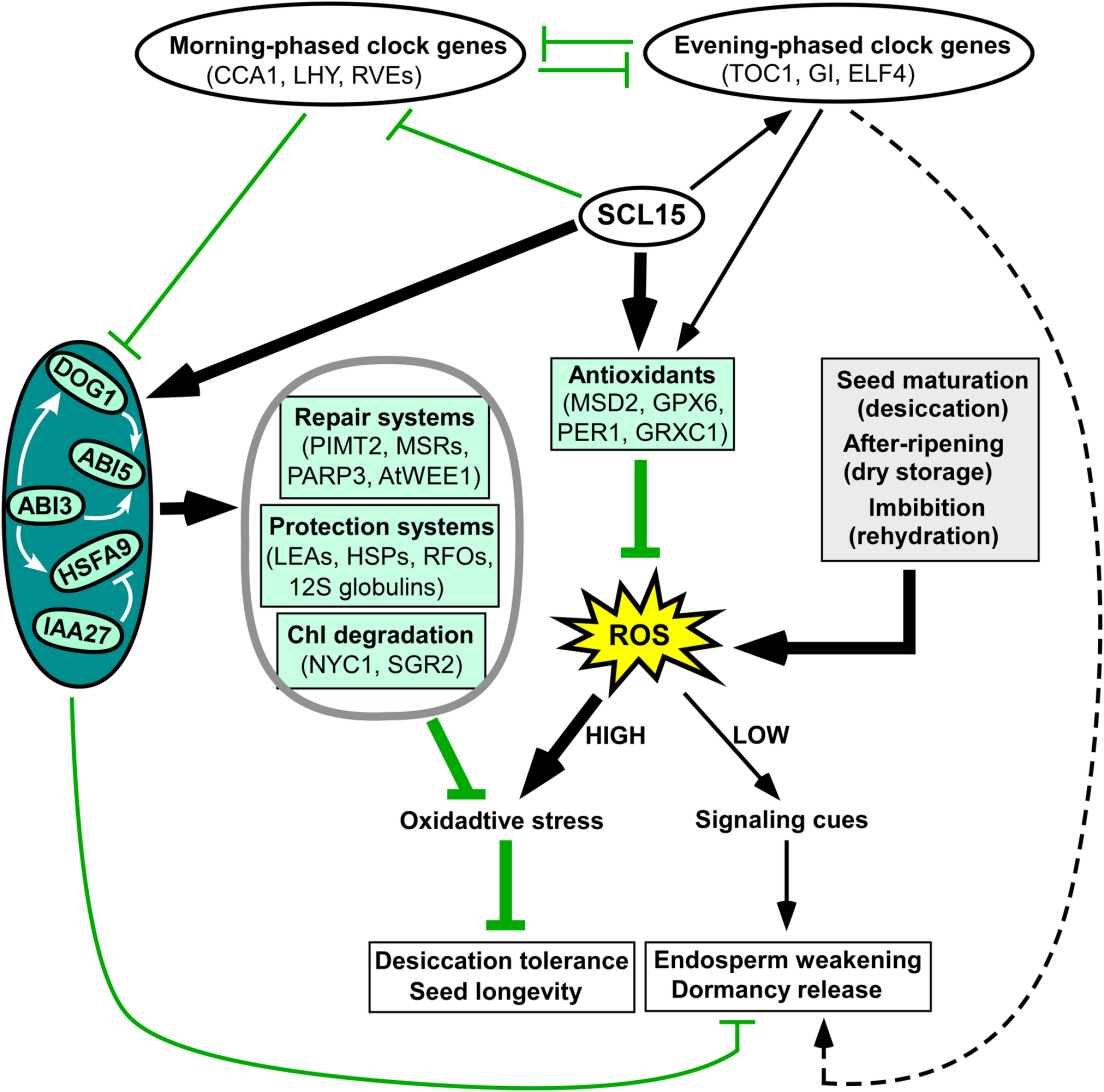
Schematic model illustrating the regulatory role of SCL15 in seed longevity. ROS exert dual roles during seed maturation and imbibition; moderate levels act as signals to promote dormancy release, whereas excessive accumulation causes oxidative stress that reduces vigor and longevity. SCL15 enhances antioxidant capacity by activating ROS‐processing genes (*MSD2*, *GPX6*), promotes chlorophyll degradation (*NYC1*, *SGR2*), and strengthens repair systems (*PIMT2*, *PARP3*, *AtWEE1*). It also stimulates the accumulation of protective molecules, including LEA proteins, HSPs, RFOs, and 12S globulins, coordinated with regulators, such as ABI3, ABI5, DOG1, HSFA9, and IAA27. Additionally, SCL15 modulates circadian networks by repressing morning‐phased genes (*CCA1*, *LHY*) and activating evening‐phased genes (*TOC1*, *GI*), which together regulate ROS homeostasis and influence longevity and dormancy. Arrows denote activation, blunt lines repression; thick bars highlight major pathways, and solid versus dashed lines indicate direct versus indirect regulation.

SCL15 thus appears to function at the intersection of seed dormancy and longevity, mediating a developmental tradeoff between these two tightly‐linked traits (Figure 9). Previous work showed that SCL15 represses primary seed dormancy by modulating circadian‐ and hormone‐mediated gene networks during maturation, suppressing morning‐phased clock genes, such as *CCA1*, *LHY*, *RVE1*, and *RVE7*, while activating evening‐phased genes, like *GI*, *LUX*, *TOC1*, and *ELF4* (Gao et al. 2025). Morning genes reinforce dormancy through ABA signaling and repression of ROS‐scavenging pathways, limiting antioxidant protection and longevity (Penfield and Hall 2009; Lai et al. 2012), whereas evening genes attenuate ABA responses and enhance ROS detoxification and DNA repair, thereby supporting seed viability (Lai et al. 2012). Through this temporal reprogramming, SCL15 coordinates auxin‐ and ABA‐mediated regulation of endosperm wall remodeling, aided by moderate ROS signaling, to promote dormancy release while simultaneously strengthening desiccation tolerance and protective systems required for longevity acquisition (Gao et al. 2025). This dual role aligns with the broader genetic tradeoff observed between dormancy and longevity in *A. thaliana* , where deep dormancy is often linked to reduced lifespan (Nguyen et al. 2012; Rehmani et al. 2022). For instance, the ABA catabolism via CYP707A2 reduces dormancy yet promotes longevity (Okamoto et al. 2006; He et al. 2014; Sano et al. 2016), whereas ABA‐deficient and LAFL mutants exhibit concurrent declines in both traits due to impaired accumulation of protective compounds (Rehmani et al. 2022). Together, these findings indicate that SCL15 positively regulates H_2_O_2_ metabolism, antioxidant defense, cellular repair and chlorophyll degradation while repressing photoactive and metabolically active pathways during maturation. Rather than focusing on deterioration processes during storage, our data support a model in which SCL15 primarily controls the acquisition of intrinsic longevity during seed development by coordinating antioxidant capacity, macromolecular protection, and metabolic quiescence prior to desiccation (Figure 9).

In conclusion, SCL15 is a critical positive regulator of seed longevity acquisition, particularly during the late stages of seed maturation when protective and repair systems are established. By integrating ABA and auxin signaling with circadian reprogramming, SCL15 enhances ROS detoxification, promotes chlorophyll degradation and activates protective (LEAs, HSPs, RFOs, SSPs) and reparative (MSRs, PIMTs, PARP3) systems, thereby reinforcing desiccation tolerance and ensuring effective longevity acquisition during seed development. These maturation‐stage functions help preserve seed viability more effectively during subsequent dry storage. Through coordinated repression of morning‐phased clock genes, activation of evening‐phased components, and maintenance of ROS homeostasis, SCL15 helps mediate the developmental tradeoff between dormancy release and longevity acquisition. Future studies identifying its direct targets and interacting partners will help establish the molecular framework through which SCL15 safeguards seed lifespan.

## Author Contributions

M.‐J.G. and D.H. designed the research. M.‐J.G., Q.C., M.H., R.Z., and C.C. performed the research and analyzed the data. M.‐J.G. and D.H. wrote the paper.

## Disclosure

Generative AI was not used in the writing of this manuscript or in the preparation of figures.

## Conflicts of Interest

The authors declare no conflicts of interest.

## Supporting information


**Figure S1:** A representative electrochromatogram of seed protein from *Arabidopsis thaliana* cv. Col from the Bio‐Rad's Experion Automated Electrophoresis under reduced conditions.
**Figure S2:** Effects of the *SCL15* mutation on plant growth and development in *Arabidopsis thaliana*.
**Figure S3:** Singular GO enrichment analysis of *SCL15*‐upregulated genes using AgriGO.
**Figure S4:** Singular GO enrichment analysis of biological processes negatively regulated by *SCL15*.
**Figure S5:** Tetrazolium‐based assessment of seed viability.
**Figure S6:** Expression of *SCL15/AtHAM4* and HAM homologs *AtHAM1/2/3* in developing *Arabidopsis thaliana* embryos (A), seeds (B), and root tissues (C), showing the unique expression patterns for *SCL15* in maturing seeds and in the vasculature.

**Table S1:** Primers used for real‐time RT‐qPCR analysis.

**Table S2:** Longevity‐associated genes with altered expression during seed maturation in *scl15‐1* and Napin:SCL15 lines as identified by RNA‐seq.

## Data Availability

The data that support the findings of this study are available from the corresponding authors upon reasonable request. The silique RNA‐seq dataset can be accessed in the NCBI GEO database as accession GSE160707.
