# Supplementary material for: SCL15 Promotes Seed Longevity Acquisition in Arabidopsis thaliana by Enhancing Antioxidant and Repair Mechanisms During Maturation
Source: Physiol Plant. 2026 May 6;178:e70907. doi: 10.1111/ppl.70907 (PMC13149782; doi:10.1111/ppl.70907)
Supplement: Supplementary file 1 — Figure S1: A representative electrochromatogram of seed protein from Arabidopsis thaliana cv. Col from the Bio‐Rad's Experion Automated Electrophoresis under reduced conditions. Figure S2: Effects of the SCL15 mutation on plant growth and development in Arabidopsis thaliana . Figure S3: Singular GO enrichment analysis of SCL15‐upregulated genes using AgriGO. Figure S4: Singular GO enrichment analysis of biological processes negatively regulated by SCL15. Figure S5: Tetrazolium‐based assessment of seed viability. Figure S6: Expression of SCL15/AtHAM4 and HAM homologs AtHAM1/2/3 in developing Arabidopsis thaliana embryos (A), seeds (B), and root tissues (C), showing the unique expression patterns for SCL15 in maturing seeds and in the vasculature. [file PPL-178-e70907-s002.pdf]

SUPPLEMENTALS

SCL15 promotes seed longevity acquisition in *Arabidopsis thaliana* by enhancing antioxidant and repair mechanisms during maturation

Ming-Jun Gao[1,*], Cathy Coutu[1], Qi Chen[2], Myrtle Harrington[1], Rong Zhou[1], and Dwayne Hegedus[1,*]

[1]Agriculture and Agri-Food Canada, Saskatoon Research Centre, 107 Science Place, Saskatoon, S7N 0X2, SK, Canada
[3]State Key Laboratory of Tea Plant Biology and Utilization, Anhui Agricultural University, Hefei, Anhui, China

*Corresponding authors: Dwayne Hegedus, Dwayne.hegedus@canada.ca; Ming-Jun Gao, ming-jun.gao@canada.ca

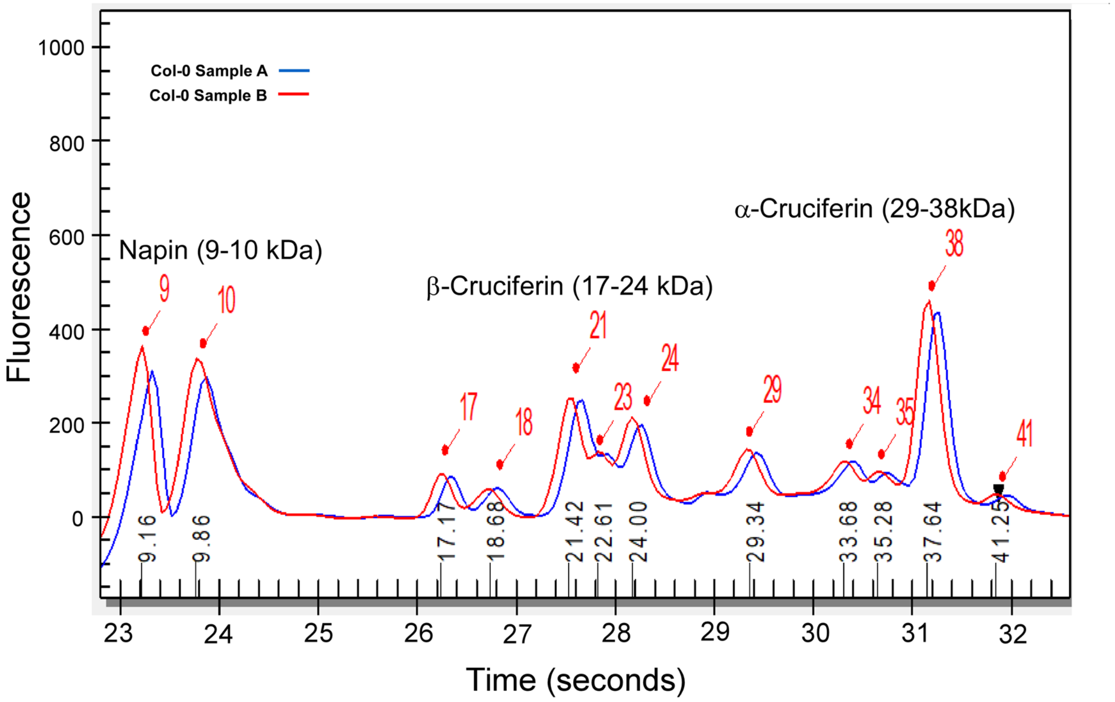

Figure S1. A representative electrochromatogram of seed protein from *A. thaliana* cv. Col from the Bio-Rad's Experion Automated Electrophoresis under reduced conditions. The Experion chip-based microfluidic electrophoresis system uses the Experion software to analyze the data. The software integrates the signals detected for the internal standard markers and sample proteins. The data is then displayed as an electrochromatogram with protein abundance being quantified based on peak area. Similar analysis was conducted for the evaluation of seed storage protein in *Camelina sativa* (Lyzenga et al. 2019).

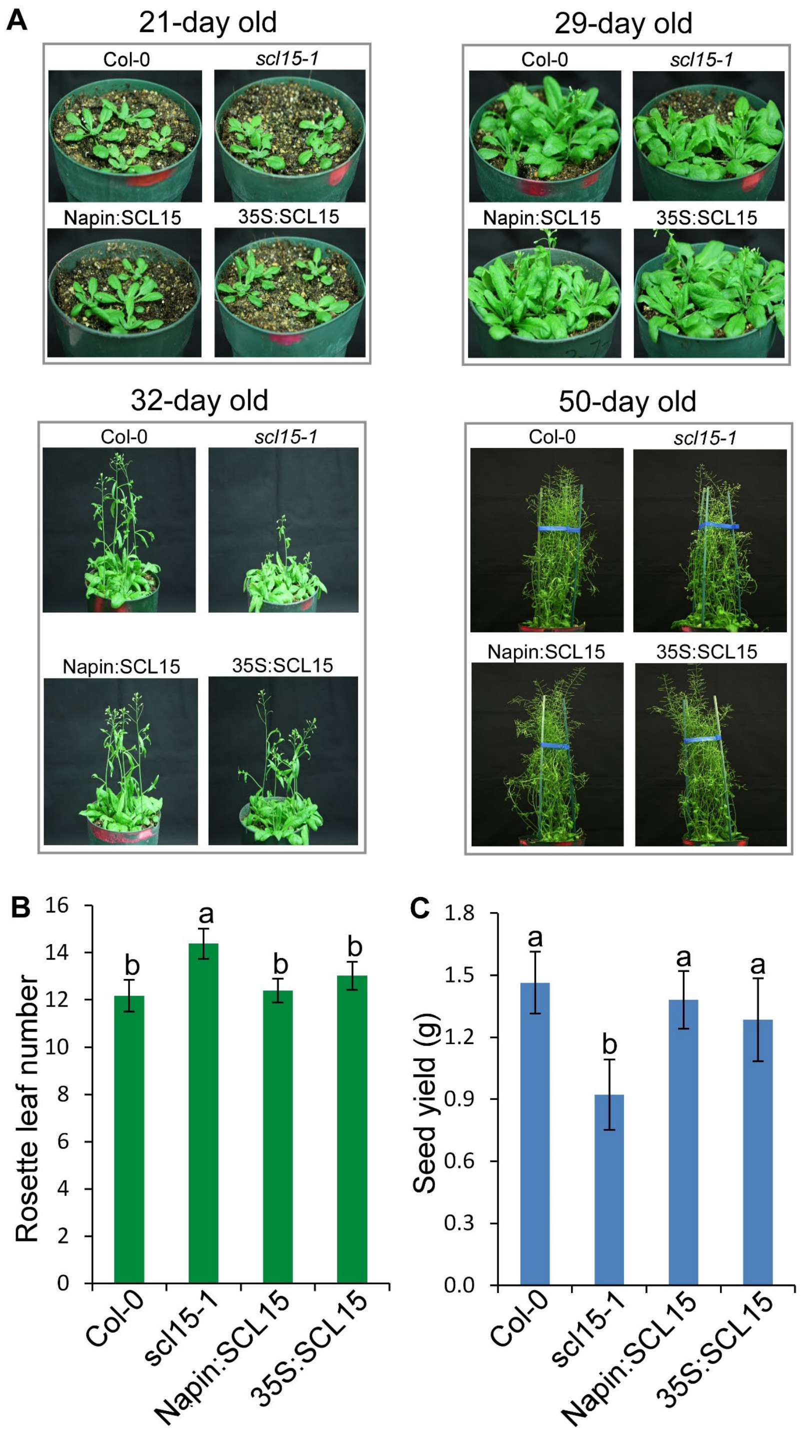

Figure S2. Effects of the *SCL15* mutation on plant growth and development in *Arabidopsis thaliana*. (**A**) Phenotypes of 21-49-day-old wild-type Col-0, *scl15-1*, Napin:SCL15, and 35S:SCL15 plants. (**B**) Rosette leaf numbers of Col-0, *scl15-1*, and Napin:SCL15 and 35S:SCL15 overexpression lines. Error bars represent mean ± SD (n = 15). (**C**) Seed yields of Col-0, *scl15-1*, Napin:SCL15, and 35S:SCL15. Error bars represent mean ± SD (n = 10). Different letters indicate significant differences determined by one-way ANOVA followed by Tukey's HSD test ($P < 0.05$).

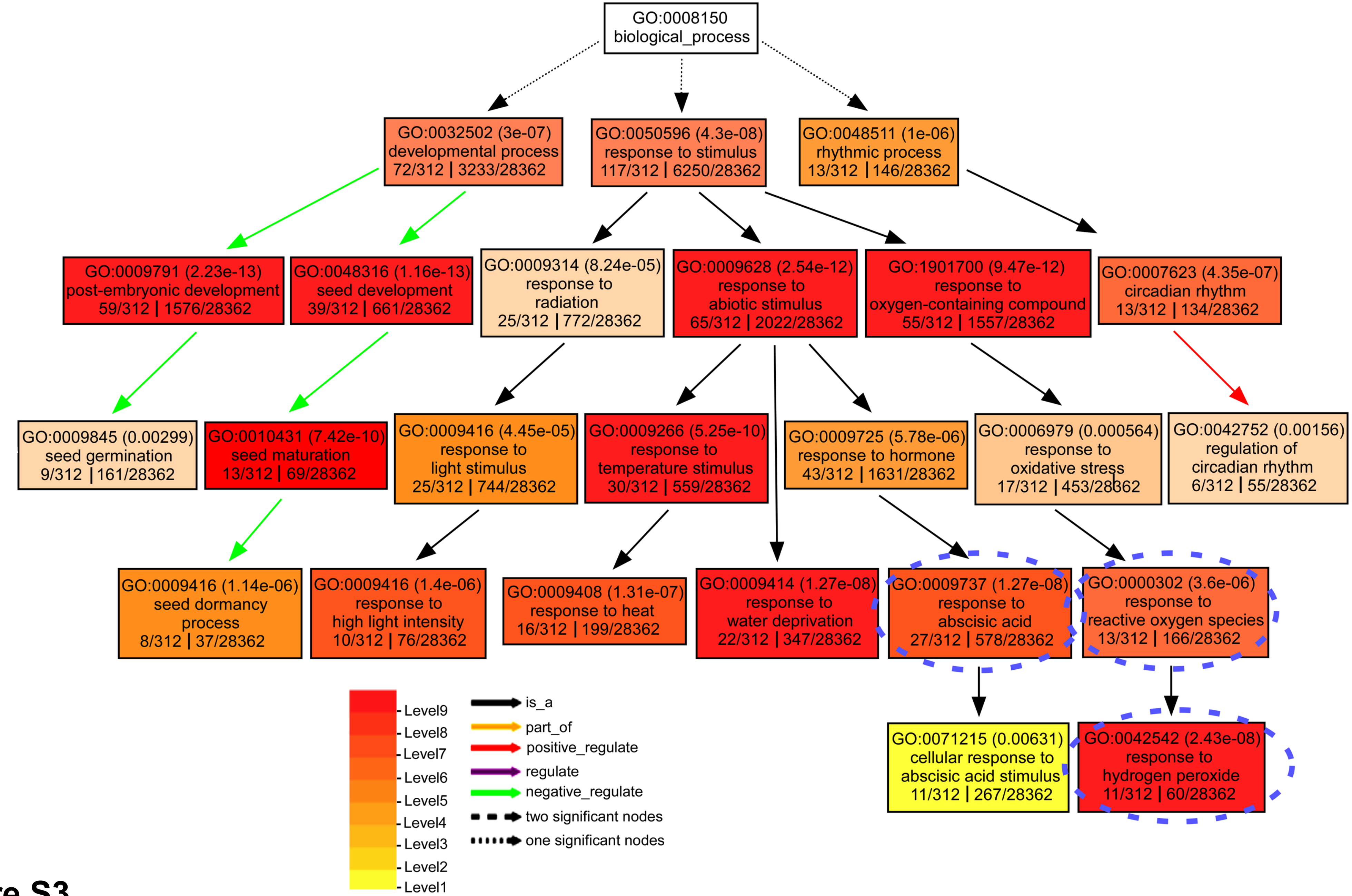
GO:0008150
biological_process
GO:0032502 (3e-07)
developmental process
72/312 | 3233/28362
GO:0050596 (4.3e-08)
response to stimulus
117/312 | 6250/28362
GO:0048511 (1e-06)
rhythmic process
13/312 | 146/28362
GO:0009791 (2.23e-13)
post-embryonic development
59/312 | 1576/28362
GO:0048316 (1.16e-13)
seed development
39/312 | 661/28362
GO:0009314 (8.24e-05)
response to
radiation
25/312 | 772/28362
GO:0009628 (2.54e-12)
response to
abiotic stimulus
65/312 | 2022/28362
GO:1901700 (9.47e-12)
response to
oxygen-containing compound
55/312 | 1557/28362
GO:0007623 (4.35e-07)
circadian rhythm
13/312 | 134/28362
GO:0009845 (0.00299)
seed germination
9/312 | 161/28362
GO:0010431 (7.42e-10)
seed maturation
13/312 | 69/28362
GO:0009416 (4.45e-05)
response to
light stimulus
25/312 | 744/28362
GO:0009266 (5.25e-10)
response to
temperature stimulus
30/312 | 559/28362
GO:0009725 (5.78e-06)
response to hormone
43/312 | 1631/28362
GO:0006979 (0.000564)
response to
oxidative stress
17/312 | 453/28362
GO:0042752 (0.00156)
regulation of
circadian rhythm
6/312 | 55/28362
GO:0009416 (1.14e-06)
seed dormancy
process
8/312 | 37/28362
GO:0009416 (1.4e-06)
response to
high light intensity
10/312 | 76/28362
GO:0009408 (1.31e-07)
response to heat
16/312 | 199/28362
GO:0009414 (1.27e-08)
response to
water deprivation
22/312 | 347/28362
GO:0009737 (1.27e-08)
response to
abscisic acid
27/312 | 578/28362
GO:0000302 (3.6e-06)
response to
reactive oxygen species
13/312 | 166/28362
GO:0071215 (0.00631)
cellular response to
abscisic acid stimulus
11/312 | 267/28362
GO:0042542 (2.43e-08)
response to
hydrogen peroxide
11/312 | 60/28362
Level9
Level8
Level7
Level6
Level5
Level4
Level3
Level2
Level1
is_a
part_of
positive_regulate
regulate
negative_regulate
two significant nodes
one significant nodes

Figure S3.

Figure S3. Singular GO enrichment analysis of *SCL15*-upregulated genes using AgriGO. GO analysis was performed on 317 genes that were downregulated in *scl15-1* and upregulated in Napin:SCL15 compared with WT. The most significantly enriched GO terms and associated biological processes are shown. GO terms previously linked to seed longevity are highlighted with dashed circles. Each box displays the GO term ID and *p*-value. The numeral pair on the left indicates the number of input genes associated with that GO term and the total number of input genes. The numeral pair on the right shows the number of genes annotated to that GO term in the TAIR10_2017 database and the total number of annotated genes. Arrows indicate pathway direction and type. Box colors represent statistical significance: yellow = $p < 0.05$, orange = $p < 1e{-5}$, and red = $p < 1e{-9}$.

Singular Gene Ontology (GO) enrichment analysis of DEGs upregulated by *SCL15* in maturing seeds using AgriGO v2.

Unclassified / Non classifié

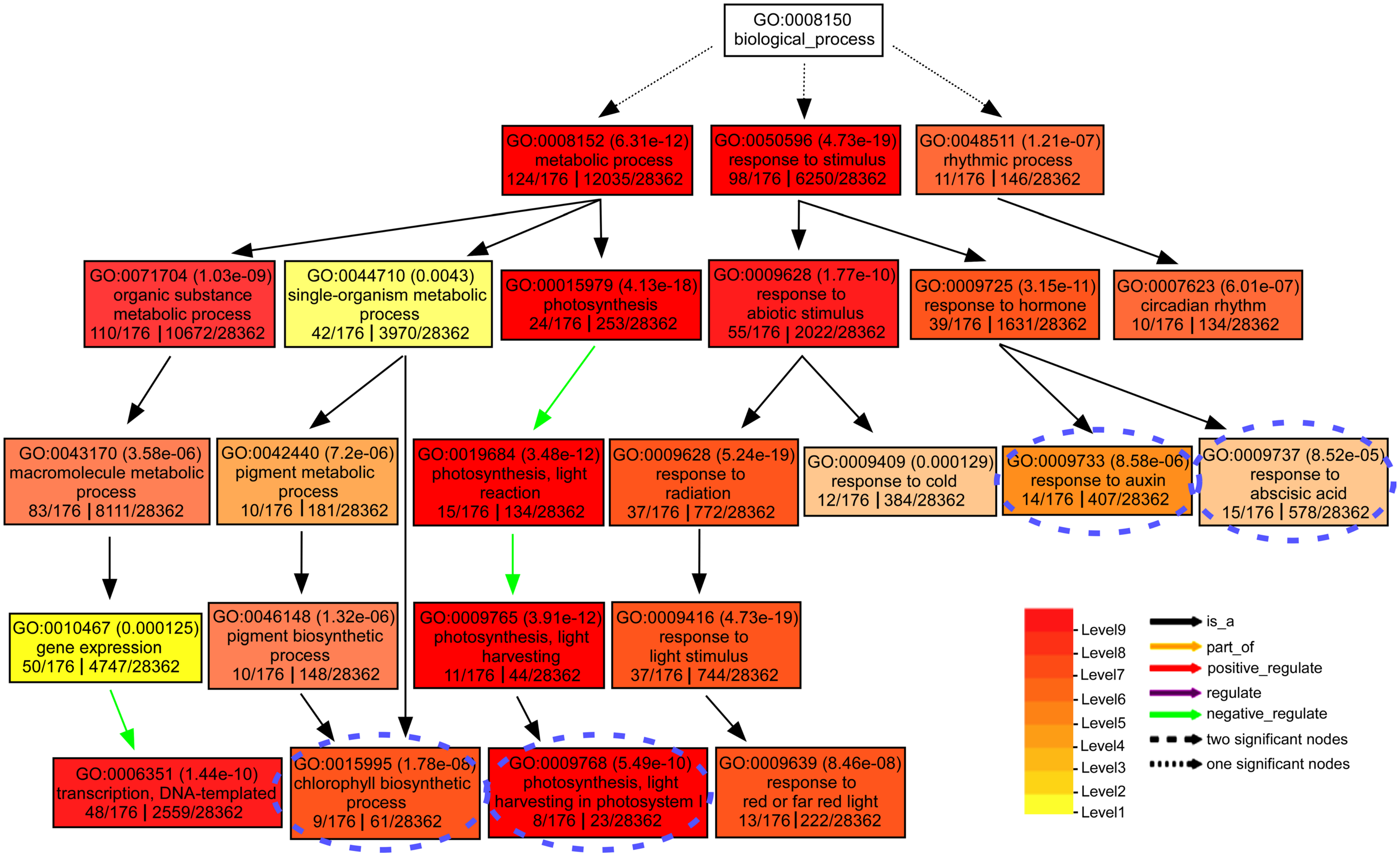

Figure S4.

Figure S4. Singular GO enrichment analysis of biological processes negatively regulated by *SCL15*. GO analysis was performed using AgriGO on 178 genes that were upregulated in *scl15-1* and downregulated in Napin:SCL15 compared with WT. The most significantly enriched GO terms and associated biological pathways are shown. GO terms previously associated with seed longevity are highlighted with dashed circles.

Unclassified / Non classifié

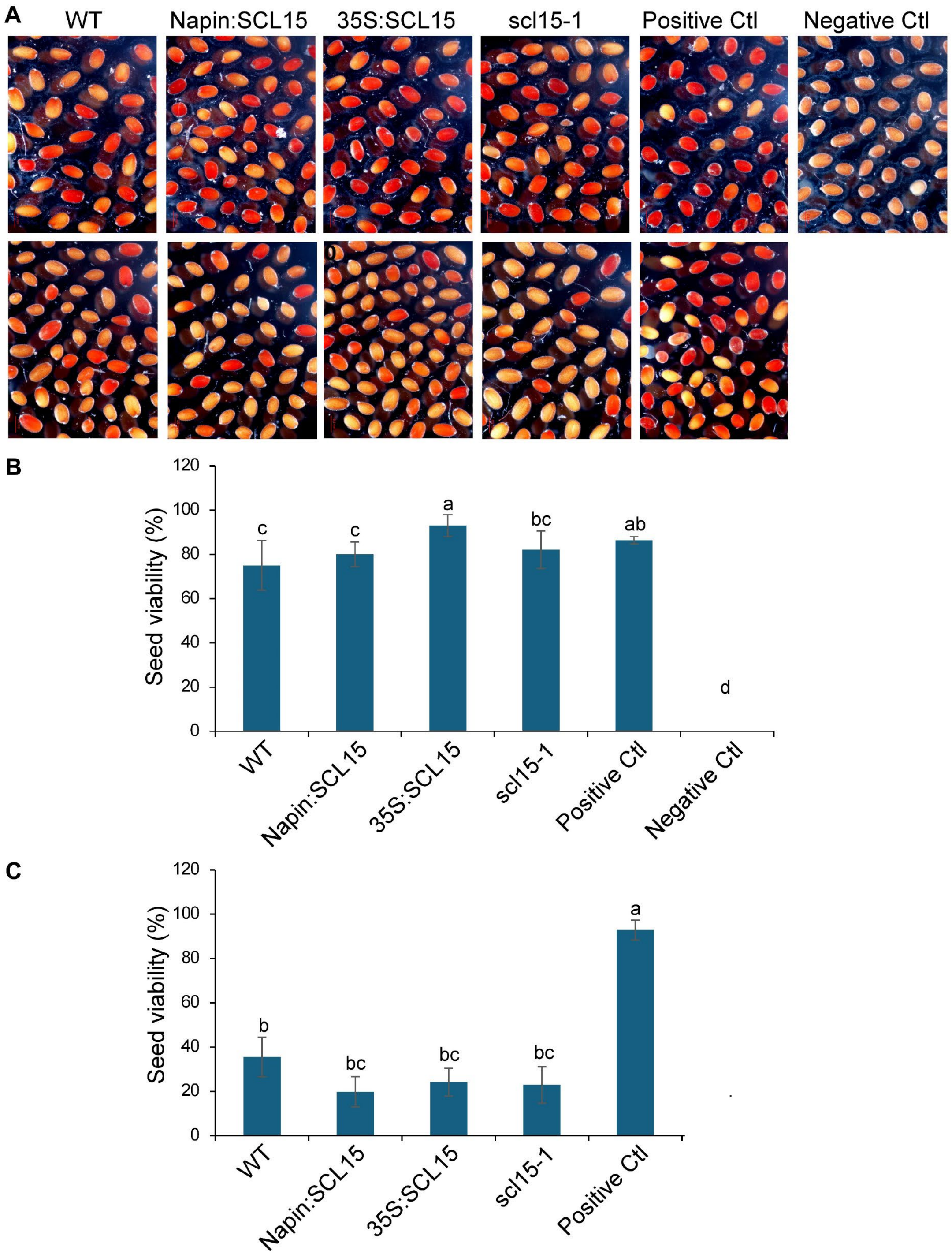

Fig. S5. Tetrazolium-based assessment of seed viability. (**A**) TTC staining of WT, *scl15-1*, and SCL15-overexpression lines (Napin:SCL15 and 35S:SCL15) after seven years (top panel) or nine years (bottom panel) of dry storage. WT seeds stored for one year were used as a positive control (Ctl), while heat-killed WT seeds stored for one year served as a negative control. (**B**) Percentage viability of seeds stored for seven years, as determined by TTC staining. (**C**) Percentage viability of seeds stored for nine years, as determined by TTC staining. Error bars represent mean ± SD ($n = 3$). Different letters indicate significant differences determined by one-way ANOVA followed by Tukey's HSD test ($P < 0.01$).

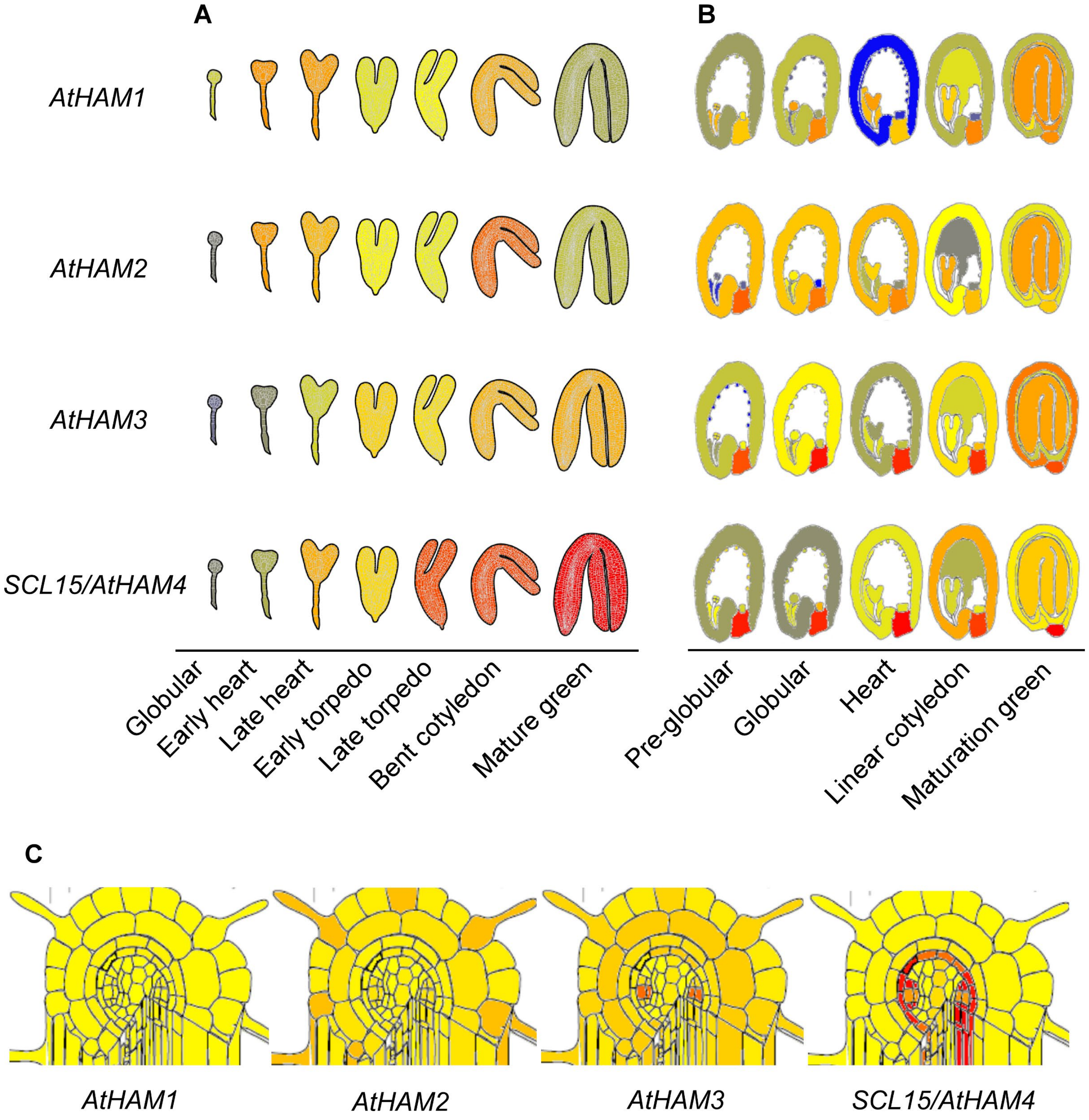

Figure S6. Expression of *SCL15/AtHAM4* and HAM homologs *AtHAM1/2/3* in developing *A. thaliana* embryos (**A**), seeds (**B**) and root tissues (**C**), showing the unique expression patterns for *SCL15* in maturing seeds and in the vasculature. Data are derived from the Arabidopsis eFP Browser (http://bar.utoronto.ca/efp/cgi-bin/efpWeb.cgi).
