# Supplementary material for: SCL15 Promotes Seed Longevity Acquisition in Arabidopsis thaliana by Enhancing Antioxidant and Repair Mechanisms During Maturation
Source: Physiol Plant. 2026 May 6;178:e70907. doi: 10.1111/ppl.70907 (PMC13149782; doi:10.1111/ppl.70907)
Supplement: Supplementary file 2 — Table S1: Primers used for real‐time RT‐qPCR analysis. [file PPL-178-e70907-s001.docx]

**Table S1. Primers used for real-time qRT-PCR analysis**

| Gene | Gene Code | Orientation | Sequence (5' - 3') |
| --- | --- | --- | --- |
| ABI3 | At3g24650 | Forward | GGAAACTGTGACGACTCTTCTGGT |
|  |  | Reverse | CTGAGGTGTCAAAGAACTCGTTGCT |
| CRC | At4g28520 | Forward | TTGACGTTCAGTTGGCTCAGCAG |
|  |  | Reverse | TGCAGATAGTCTCCTCAAGGCCG |
| CRA1 | At5g44120 | Forward | CAGGAACCACTTGTCATCGTCAG |
|  |  | Reverse | CTCTCGTCCTTGTAGCCATACTTG |
| LEC1 | At1g21970 | Forward | ACCAGCTCAGTCGTAGTAGCC |
|  |  | Reverse | GTGAGACGGTAAGGTTTTACGCATGAT |
| Oleo2 | At5g40420 | Forward | GTTACGGTGGTGGCGGTTACAA |
|  |  | Reverse | CAACCATTAAGCCGATCACCGA |
| RAB18 | At5g66400 | Forward | CATGGCGTCTTACCAGAACCGTC |
|  |  | Reverse | TCCTCCCATCGGATTTCCGTACT |
| d-VPE | At3g20210 | Forward | AAAACCCAATTGCTGAACGA |
|  |  | Reverse | AAACCGCCTTTTCGGAGTAT |
| a-TIP | At1g73190 | Forward | CGACTCATAGTTGGGGCAAA |
|  |  | Reverse | TAAGGGCGGCTAAAGCACTA |
| ACT2 | [At3g18780](http://www.arabidopsis.org/servlets/TairObject?id=39062&type=locus) | Forward | GTTTCGCCACGTGGAATCCTCT |
|  |  | Reverse | GACTTCTGGGCATCTGAATCTCTCA |
| Ef-1a | At5g60390 | Forward | TGAGCACGCTCTTCTTGCTTTCA |
|  |  | Reverse | GGTGGTGGCATCCATCTTGTTACA |
| ABI5 | At2g36270 | Forward | GGAGAGATGACACTTGAGGATTT |
|  |  | Reverse | TGGTTCGGGTTTGGATTAGG |
| EM1 | At3g51810 | Forward | TAGGGCACGAGGGTTATCA |
|  |  | Reverse | TCCTCCTTTACGTCCCATCT |
| NYC1 | At4g13250 | Forward | GGGAAATGGGTCGTGGGATT |
|  |  | Reverse | AGCTTCTCAACGTCTTCGGG |
| SGR2 | At4g11910 | Forward | AGATTGTTTGGACCGGCGAT |
|  |  | Reverse | CCGTCGGAAAGCAACACTTG |
| PSAN | At5g64040 | Forward | CTCTTCCTTGCTCACTCACCA |
|  |  | Reverse | GCCTTCGCATTCCAAAGCAA |
| Lhcb1.3 | At1g29930 | Forward | GACTGTTGCCAAGCCAAAGG |
|  |  | Reverse | GAATGCCTCTGGGTCGGTAG |
| Lhcb2.1 | At2g05100 | Forward | TCAAGCCCTCCAACGAACTC |
|  |  | Reverse | GTTCAATGGGTCGAACGCAC |
