# Supplementary material for: SCL15 Promotes Seed Longevity Acquisition in Arabidopsis thaliana by Enhancing Antioxidant and Repair Mechanisms During Maturation
Source: Physiol Plant. 2026 May 6;178:e70907. doi: 10.1111/ppl.70907 (PMC13149782; doi:10.1111/ppl.70907)
Supplement: Supplementary file 3 — Table S2: Longevity‐associated genes with altered expression during seed maturation in scl15‐1 and Napin:SCL15 lines as identified by RNA‐seq. [file PPL-178-e70907-s003.docx]

**Table S2.** Longevity-associated genes with altered expression during seed maturation in *scl15-1* and Napin:SCL15 lines, as identified by RNA-seq

| Gene Name | Locus Identifier (AGI) | | | Expression (*scl15-1* vs Col-0) | | Expression (*Napin:SCL15* vs Col-0) | | Molecular Function in Seed Longevity | | | |  |
| --- | --- | --- | --- | --- | --- | --- | --- | --- | --- | --- | --- | --- |
| Antioxidants | | | | | | | | | | | | |
| *MSD2** | AT3G56350 | | | -2.054 | | 2.224 | | Encoding a superoxide dismutase (SOD) catalyzing the conversion of superoxide into oxygen and hydrogen peroxide (Chen et al., 2022; Lee et al., 2022). | | | |  |
| *GPX1* | AT2G25080 | | | 1.516 | | -1.588 | | Encoding GLUTATHIONE PEROXIDASE 1 | | | |  |
| *GPX6* | AT4G11600 | | | -1.919 | | 1.526 | | Encoding a glutathione peroxidase detoxifying H_2_O_2_ and organic hydroperoxides (Belta et al., 2015; Attacha et al., 2017) | | | |  |
| *GPX6* | AT4G31870 | | | -3.906 | | 1.026 | | Encoding GLUTATHIONE PEROXIDASE 7 and mainly expressed in the seed endosperm | | | |  |
| *PER1* | AT1G48130 | | | -1.545 | | 1.755 | | Encoding a peroxide reductase detoxifying hydroxyl radicals (OH^•^) in addition to reduce H_2_O_2_ (Chen et al., 2016; Wang et al., 2018) | | | |  |
| *PER13* | AT1G77100 | | | -1.931 | | 2.103 | | Encoding a peroxide reductase detoxifying hydroxyl radicals (OH^•^) in addition to reduce H_2_O_2_ (Chen et al., 2016; Wang et al., 2018) | | | |  |
| *PER59* | AT5G19890 | | | -94.875 | | 1.448 | | Encoding a peroxidase protein | | | |  |
| *GLYI4* | AT1G15380 | | | -2.389 | | -1.040 | | Encoding GLYOXYLASE I 4 | | | |  |
| *GLYI8* | AT2G28420 | | | -2.049 | | 1.783 | | Encoding a glyoxalase detoxifying reactive carbonyl specie (Schmitz et al., 2018) | | | |  |
| *GRXC1* | AT5G63030 | | | -1.669 | | 1.985 | | Encoding an oxidoreductase involved in the maintenance of cellular redox homeostasis and protection against protein oxidative damage (Cheng et al., 2006; Riondet et al., 2012; Jung et al., 2018) | | | |  |
| *GSTU3* | AT2G29470 | | | -12.169 | | 1.593 | | Encoding glutathione S-transferase tau 3 | | | |  |
| *GSTU4* | AT2G29460 | | | -1.608 | | 1.504 | | Encoding a glutathione transferase involved in the detoxification and antioxidant response (Zhang et al., 2017; Wu et al., 2024). | | | |  |
| *DOX1* | AT3G01420 | | | -2.304 | | 1.515 | | Encoding ALPHA-DIOXYGENASE 1 | | | |  |
| *AtFER1* | AT3G11050 | | | -3.278 | | 2.053 | | Encoding FERRITIN 2 | | | |  |
| Chlorophyll metabolism and photosynthesis | | | | | | | | | | |  |  |
| *SGR2*** | AT4G11910 | | | -1.795 | | 1.523 | | Encoding STAY-GREEN2 contributing embryo-specific degreening (Delmas et al., 2013). | | | |  |
| *NYC1*** | AT4G13250 | | | -1.898 | | 1.206 | | Encoding NON-YELLOW COLORING1 catalyzing the first step of chlorophyll catabolism (Nakajima et al., 2012) | | | |  |
| *Lhcb1.1* | AT1G29920 | | | 2.451 | | -2.130 | | Encoding light-harvesting complex (LHC) b1.1 primarily associated with Photosystem (PS) II (Standfuss et al., 2005; Pietrzykowska et al., 2014) | | | |  |
| *Lhcb1.2* | AT1G29910 | | | 2.403 | | -1.682 | | Encoding LHC b1.2 primarily associated with PSII (Standfuss et al., 2005; Pietrzykowska et al., 2014) | | | |  |
| *Lhcb1.3*** | AT1G29930 | | | 1.746 | | -1.449 | | Encoding LHC b1.3 primarily associated with PSII (Standfuss et al., 2005; Pietrzykowska et al., 2014) | | | |  |
| *PSAN*** | AT5G64040 | | | 1.909 | | -1.652 | | Encoding PSI Subunit N (PSI-N) (Rolo et al., 2024). | | | |  |
| *Lhcb2.1*** | AT2G05100 | | | 1.682 | | -2.271 | | Encoding LHC b2.1 primarily associated with PSII (Standfuss et al., 2005; Pietrzykowska et al., 2014) | | | |  |
| *Lhcb2.2* | AT2G05070 | | | 1.816 | | -1.843 | | Encoding LHC b2.2 primarily associated with PSII (Standfuss et al., 2005; Pietrzykowska et al., 2014) | | | |  |
| *Lhcb3* | AT5G54270 | | | 1.844 | | -1.518 | | Encoding LHC b3 primarily associated with PSII (Standfuss et al., 2005; Pietrzykowska et al., 2014) | | | |  |
| *ELIP1* | AT3G22840 | | | -5.111 | | 1.635 | | Encoding EARLY LIGHT-INDUCABLE PROTEIN 1 | | | |  |
| *ELIP2* | AT4G14690 | | | 1.742 | | -1.656 | | Encoding EARLY LIGHT-INDUCABLE PROTEIN 2 | | | |  |
| *GUN5/CHLH/ABAR* | AT5G13630 | | | 1.605 | | -1.867 | | Encoding GENOMES UNCOUPLED 5/ H SUBUNIT OF MG-CHELATASE/ ABA-BINDING PROTEIN | | | |  |
| *CHLI1* | AT4G18480 | | | 1.542 | | -1.541 | | Encoding CHLI SUBUNIT OF MG-CHELATASE | | | |  |
| Protein and DNA repair factors | | | | | | | | | | | | |
| *PIMT2** | AT5G50240 | | | -2.585 | | 2.580 | | Encoding a protein repairing enzyme Protein l-isoaspartyl Methyltransferase involved in seed desiccation tolerance and longevity (Ogé et al., 2008; Verma et al., 2013; Petla et al., 2016; Kamble & Majee, 2022). | | | |  |
| *MSRB6* | AT4G04840 | | | -1.535 | | 1.546 | | Encoding a protein repairing enzyme Methionine Sulfoxide Reductase involved in preserving seed vigor and longevity (Chatelain et al., 2013; Hazra et al., 2022; Cai et al., 2023). | | | |  |
| *PARP3* | AT5G22470 | | | -2.446 | | 2.389 | | Encoding a Poly (ADP-ribose) Polymerase involved in base damage repair (Boehler et al., 2011; Rissel et al., 2014). | | | |  |
| *AtWEE1* | AT1G02970 | | | 1.960 | | -4.145 | | Encoding a conserved WEE1 homolog, a key player of DNA damage response via cell cycle checkpoint pathway (Pedroza‐Garcia et al., 2022). | | | |  |
| *CYCD3;2* | AT5G67260 | | | 1.510 | | -1.568 | | Encoding plant‐specific D3‐type cyclin essential for promoting cell division and required for cell-cycle-regulated DNA damage repair (Collins et al., 2012; Weimer et al., 2016). | | | |  |
| *CYCP4;1* | AT2G44740 | | | 2.539 | | -2.478 | | Encoding cyclin P4;1 involved in promoting cell division and repairing cell-cycle-regulated DNA damage (Weimer et al., 2016). | | | |  |
| *CDKB2;2* | AT1G20930 | | | 1.756 | | -2.742 | | Encoding cyclin-dependent kinase B2;2 involved in SOG1-regulated DNA damage response (Ogita et al., 2018). | | | |  |
| *CYCA1;1* | AT1G44110 | | | 2.648 | | -2.359 | | Encoding cyclin A1;1 | | | |  |
| *CYCB1;2* | AT5G06150 | | | 2.690 | | -2.643 | | Encoding cyclin B1;2 | | | |  |
| *CYCB1;4* | AT2G26760 | | | 2.078 | | -1.526 | | Encoding cyclin B1;4 | | | |  |
| *ENODL14* | AT2G25060 | | | 2.867 | | -2.999 | | Encoding early nodulin-like protein 14 | | | |  |
| Protection proteins | | | | | | | | | | | | |
| *AtSTS* | AT4G01970 | | | -1.548 | | 1.687 | | Encoding a Stachyose Synthase with some Raffinose Synthase capacity correlated with seed vigor and longevity (Li et al., 2017). | | | |  |
| *AGAL2* | AT5G08370 | | | 1.586 | | -4.120 | | Encoding a α-galactosidase involved in hydrolyzation of raffinose family oligosaccharides (Shabalin et al., 2002). | | | |  |
| *AtEM1**^/^** | AT3G51810 | | | -6.679 | | 2.535 | | Encoding a group 1 LEA having similar function with *AtEM6* | | | |  |
| *AtEM6** | AT2G40170 | | | -24.683 | | 2.379 | | Encoding a group 1 LEA regulated by ABI5 and involved in the control of desiccation tolerance (Nakamura et al., 2001; Manfre et al., 2009). | | | |  |
| *LEA4-1* | AT1G32560 | | | -1.613 | | 1.768 | | Encoding a group 4 LEA possessing chaperone-like activity to protect enzyme activities in response to dehydration stress (Cuevas-Velazquez et al., 2016; Rendón-Luna et al., 2024). | | | |  |
| *LEA4-2/LEA18** | AT2G35300 | | | -3.588 | | 1.984 | | Having similar function with *LEA4-1* | | | |  |
| *LEA4-5** | AT5G06760 | | | -3.626 | | 2.173 | | Having similar function with *LEA4-1* | | | |  |
| *LEA5-5* | AT5G53260 | | | -10.382 | | 3.339 | | Encoding a cytosol and nucleus dual-localized seed maturation protein | | | |  |
| *LEA13* | AT2G18340 | | | -5.615 | | 2.697 | | Encoding a LEA family protein | | | |  |
| *LEA14* | AT2G21490 | | | -1.541 | | 1.905 | | Encoding a cytosol and nucleus dual-localized dehydrin family protein | | | |  |
| *LEA25* | AT2G42560 | | | -7.784 | | 2.532 | | Encoding a cytosol-localized LEA family protein | | | |  |
| *LEA30* | AT1G22600 | | | -2.476 | | 1.957 | | Encoding a LEA family protein | | | |  |
| *LEA29* | AT3G15670 | | | -3.838 | | 2.095 | | Encoding a nucleus-localized LEA family protein | | | |  |
| *LEA45* | AT4G39130 | | | -3.337 | | 3.000 | | Encoding a cytosol and nucleus dual-localized dehydrin family protein | | | |  |
| *RAB18**^/^** | AT3G22490 | | | -4.096 | | 2.273 | | Encoding an ABA- and drought-induced glycine-rich dehydrin protein RESPONSIVE TO ABA 18 | | | |  |
| *RAB28** | AT3G22490 | | | -19.248 | | 2.616 | | Encoding RESPONSIVE TO ABA 28 | | | |  |
| *M10** | AT2G41280 | | | -17.149 | | 1.560 | | Encoding LEA protein M10 | | | |  |
| *SSLEA* | AT3G17520 | | | -3.172 | | 2.152 | | Encoding a LEA family protein | | | |  |
| *XERO1** | AT3G50980 | | | -2.528 | | 1.884 | | Encoding a cytosol and nucleus dual-localized dehydrin family protein XERO1 | | | |  |
| *XERO2** | AT3G50970 | | | -2.473 | | 1.542 | | Encoding a cytosol and nucleus dual-localized dehydrin family protein XERO2 | | | |  |
| *ATECP31* | AT3G22500 | | | -15.457 | | 1.931 | | Encoding a cytosol and nucleus dual-localized seed maturation protein | | | |  |
| *HSFA9* | AT5G54070 | | | -10.430 | | 2.509 | | Encoding Heat Shock Transcription Factor A9 playing a central role in seed longevity and desiccation tolerance under the control of ABI3 and IAA27 (Zinsmeister et al., 2020; Wang et al., 2024). | | | |  |
| *HSFA1E* | AT3G02990 | | | -1.713 | | 1.420 | | Encoding Heat Shock Transcription Factor A1E functioning as the master regulator of heat stress response and participating as important components in oxidative stress (Nishizawa-Yokoi et al., 2011; Liu et al., 2011). | | | |  |
| *HSFA2* | AT2G26150 | | | 1.835 | | -1.506 | | Encoding Heat Shock Transcription Factor A2 | | | |  |
| *HSP17.4** | AT3G46230 | | | -9.423 | | 2.608 | | Encoding Heat Shock Protein 17.4 regulated by HSFA9 and ABI3 and used as a molecular marker for oxidative stress (Sewelam et al., 2019; Zinsmeister et al., 2020). | | | |  |
| *HSP17.4B* | AT1G54050 | | | -3.353 | | 1.553 | | Encoding HSP20-like chaperones superfamily protein | | | |  |
| *HSP17.6* | AT5G12030 | | | -9.159 | | 2.037 | | Having similar function with *HSP17.4* | | | |  |
| *HSP17.6B* | AT2G29500 | | | -2.221 | | 1.758 | | Encoding HSP20-like chaperones superfamily protein | | | |  |
| *HSP17.6C** | AT1G53540 | | | -6.513 | | 2.229 | | Encoding HSP20-like chaperones superfamily protein | | | |  |
| *HSP101* | AT1G74310 | | | -2.782 | | 1.723 | | Having similar function with *HSP17.4* | | | |  |
| *HSP23.6* | AT4G25200 | | | -7.226 | | 2.462 | | Encoding mitochondrion-localized small heat shock protein 23.6 | | | |  |
| *HSP26.5** | AT1G52560 | | | -3.457 | | 1.962 | | Encoding HSP20-like chaperones superfamily protein | | | |  |
| *HSP70-5** | AT1G16030 | | | -9.875 | | 2.303 | | Encoding heat shock protein 70B | | | |  |
| *HSP70-8** | AT2G32120 | | | -6.535 | | 2.044 | | Encoding heat-shock protein 70T-2 | | | |  |
| *HSP70-10* | AT5G09590 | | | -1.793 | | 1.526 | | Encoding mitochondrial HSO70 2 | | | |  |
| *HSP81-1* | AT5G52640 | | | -1.692 | | 1.587 | | Encoding a cytosolic heat shock protein AtHSP90.1 | | | |  |
| *MED37C* | AT3G12580 | | | -1.855 | | 1.550 | | Encoding heat shock protein 70 | | | |  |
| *CRA1***/*** | AT5G44120 | | | -1.532 | | 1.924 | | Encoding 12S globulin Cruciferin A being described as a primary target for oxidation in seeds and showing severe phenotypes for seed longevity after mutation (Sano et al., 2016). | | | |  |
| *CRB* | AT1G03880 | | | -1.516 | | 1.780 | | Encoding 12S globulin Cruciferin B having similar function with *CRA* | | | |  |
| *CRC*** | AT4G28520 | | | -1.522 | | 2.089 | | Encoding 12S globulin Cruciferin C having similar function with *CRA* | | | |  |
| ABA and auxin signaling factors | | | | | | | | | |  |  |  |
| *CYP707A1* | | AT4G19230 | -1.522 | | 1.690 | | Encoding a 8'-hydroxylase CYP707A1  for ABA catabolism | |  |  |  |  |
| *CYP707A2* | | AT2G29090 | -1.779 | | 1.524 | | Encoding a 8'-hydroxylase CYP707A2 for ABA catabolism, a positive regulator of seed longevity (Okamoto et al. 2006; He et al. 2014; Sano et al. 2016). | |  |  |  |  |
| *ABI3***/*** | | AT3G24650 | Down | | Up | | Encoding ABA INSENSITIVE 3 involved in the positive regulation of seed longevity (Clerkx et al., 2004; Liu et al., 2013). | |  |  |  |  |
| *ABI5**^/^** | | AT2G36270 | -4.591 | | 2.248 | | Encoding ABA INSENSITIVE 5 involved in the positive regulation of seed longevity (Clerkx et al., 2004; Liu et al., 2013). | |  |  |  |  |
| *DOG1** | | AT5G45830 | -1.640 | | 1.998 | | Encoding DELAY OF GERMINATION 1 involved in the positive regulation of seed longevity (Bentsink et al., 2006; Dekkers et al., 2016). | |  |  |  |  |
| *UGT74E2* | | AT1G05680 | -1.775 | | 1.521 | | Encoding Uridine diphosphate glycosyltransferase 74E2, an integrator of H_2_O_2_ and auxin signaling and a positive regulator of germination under stress conditions (Wang et al., 2020). | |  |  |  |  |
| *AtRAV1* | | AT1G13260 | 1.545 | | -1.509 | | Encoding RELATED TO ABI3/VP1 1 involved in the negative regulation of seed viability and germination (Shin and Nam, 2018). | |  |  |  |  |
| *IAA1* | | AT4G14560 | 1.532 | | -2.422 | | Encoding AUX/IAA PROTEIN 1 upregulated in seeds by accelerated aging treatment (Wang et al., 2022) | |  |  |  |  |
| *IAA17* | | AT1G04250 | 2.638 | | -1.545 | | Encoding AUX/IAA PROTEIN 17 downregulated in seeds by accelerated aging treatment (Wang et al., 2022) | |  |  |  |  |
| *IAA27* | | AT4G29080 | 1.602 | | -1.536 | | Encoding AUX/IAA PROTEIN 27 whose ortholog HaIAA27 negatively regulates seed longevity by repression of the *HaHSFA9* (Carranco et al., 2010). | |  |  |  |  |

*****Validated previously by RT-qPCR analysis (Gao et al., 2025)

**Validate by real-time RT-qPCR analysis in the present study

***Validated previously by RT-qPCR analysis (Gao et al., 2015)

**References**

Attacha S., Solbach D., Bela K., Moseler A., Wagner S., Schwarzländer M., et al. (2017) Glutathione peroxidase‐like enzymes cover five distinct cell compartments and membrane surfaces in Arabidopsis thaliana. *Plant, Cell Environ.* 40: 1281-1295.

Cheng N.-H., Liu J.-Z., Brock A., Nelson R.S. and Hirschi K.D. (2006) AtGRXcp, an Arabidopsis chloroplastic glutaredoxin, is critical for protection against protein oxidative damage. *J. Biol. Chem.* 281: 26280-26288.

Dekkers B.J., He H., Hanson J., Willems L.A., Jamar D.C., Cueff G., et al. (2016) The Arabidopsis DELAY OF GERMINATION 1 gene affects ABSCISIC ACID INSENSITIVE 5 (ABI 5) expression and genetically interacts with ABI 3 during Arabidopsis seed development. *The Plant Journal* 85: 451-465.

Gao, M.-J., Chen, Q., Coutu, C., Fu, F., Yu, B., Li, X., Chen, Z.J., Hegedus, D. (2025) SCL15 regulates seed dormancy release through integration of circadian clock, hormones and cell-wall remodeling in Arabidopsis. *Physiol. Plant.* 177:e70467.

Gao, M.-J., Li, X., Huang, J., Gropp, G.M., Gjetvaj, B., Lindsay, D.L., Wei, S., Coutu, C., et al. (2015) SCARECROW-LIKE15 interacts with HISTONE DEACETYLASE19 and is essential for repressing the seed maturation programme. *Nat. Commun.* 6:7243.

Jung J.-Y., Ahn J.H. and Schachtman D.P. (2018) CC-type glutaredoxins mediate plant response and signaling under nitrate starvation in Arabidopsis. *BMC Plant Biol.* 18: 1-13.

Kamble N.U. and Majee M. (2022) ABI transcription factors and PROTEIN L-ISOASPARTYL METHYLTRANSFERASE module mediate seed desiccation tolerance and longevity in Oryza sativa. *Development* 149: dev200600.

Lee J., Chen H., Lee G., Emonet A., Kim S.G., Shim D., et al. (2022) MSD2‐mediated ROS metabolism fine‐tunes the timing of floral organ abscission in Arabidopsis. *New Phytol.* 235: 2466-2480.

Nakamura S., Lynch T.J. and Finkelstein R.R. (2001) Physical interactions between ABA response loci of Arabidopsis. *The Plant Journal* 26: 627-635.

Nishizawa-Yokoi A., Nosaka R., Hayashi H., Tainaka H., Maruta T., Tamoi M., et al. (2011) HsfA1d and HsfA1e involved in the transcriptional regulation of HsfA2 function as key regulators for the Hsf signaling network in response to environmental stress. *Plant and Cell Physiology* 52: 933-945.

Ogita N., Okushima Y., Tokizawa M., Yamamoto Y.Y., Tanaka M., Seki M., et al. (2018) Identifying the target genes of SUPPRESSOR OF GAMMA RESPONSE 1, a master transcription factor controlling DNA damage response in Arabidopsis. *The Plant Journal* 94: 439-453.

Sewelam N., Kazan K., Hüdig M., Maurino V.G. and Schenk P.M. (2019) The AtHSP17. 4C1 gene expression is mediated by diverse signals that link biotic and abiotic stress factors with ROS and can be a useful molecular marker for oxidative stress. *International journal of molecular sciences* 20: 3201.

Shin H.-y. and Nam K.H. (2018) RAV1 negatively regulates seed development by directly repressing MINI3 and IKU2 in Arabidopsis. *Molecules and cells* 41: 1072.

Wang T., Li P., Mu T., Dong G., Zheng C., Jin S., et al. (2020) Overexpression of UGT74E2, an Arabidopsis IBA glycosyltransferase, enhances seed germination and modulates stress tolerance via ABA signaling in rice. *International Journal of Molecular Sciences* 21: 7239.

Wu R., Chen B., Jia J. and Liu J. (2024) Relationship between Protein, MicroRNA Expression in Extracellular Vesicles and Rice Seed Vigor. *International Journal of Molecular Sciences* 25: 10504.

ZHANG X., TAO L., QIAO S., DU B.-h. and GUO C.-h. (2017) Roles of glutathione S-transferase in plant tolerance to abiotic stresses. *China Biotechnology* 37: 92-98.
